# An investigation to elucidate the factors dictating the crystal structure of seven ammonium carboxyl­ate mol­ecular salts

**DOI:** 10.1107/S2056989017017856

**Published:** 2018-04-17

**Authors:** Jacques Blignaut, Andreas Lemmerer

**Affiliations:** a Molecular Sciences Institute, School of Chemistry, University of the Witwatersrand, Private Bag, PO WITS, 2050, Johannesburg, South Africa

**Keywords:** crystal structure, ammonium carboxyl­ate salts, graph set, hydrogen bonding

## Abstract

The crystal structures of seven ammonium carboxyl­ate salts are reported, namely (*RS*)-1-phenyl­ethan-1-aminium isonicotinate, C_8_H_12_N^+^·C_6_H_4_N_1_O_2_^−^, (I), (*RS*)-1-phenyl­ethan-1-aminium flurbiprofenate [or 2-(3-fluoro-4-phenyl­phen­yl)propano­ate], C_8_H_12_N^+^·C_15_H_12_FO_2_^−^, (II), (*RS*)-1-phenyl­ethan-1-aminium 2-chloro-4-nitro­benzoate, C_8_H_12_N^+^·C_7_H_3_ClNO_4_^−^, (III), (*RS*)-1-phenyl­ethan-1-aminium 4-iodo­benzoate, C_8_H_12_N^+^·C_7_H_4_IO_2_^−^, (IV), (*S*)-1-cyclo­hexyl­ethan-1-aminium 2-chloro-4-nitro­benzoate, C_8_H_18_N^+^·C_7_H_3_ClNO_4_^−^, (V), 2-(cyclo­hex-1-en-1-yl)ethan-1-aminium 4-bromo­benzoate, C_8_H_16_N^+^·C_7_H_4_BrO_2_^−^, (VI), and (*S*)-1-cyclo­hexyl­ethan-1-aminium 4-bromo­benzoate, C_8_H_18_N^+^·C_7_H_4_BrO_2_^−^, (VII). Salts (II) to (VII) feature three N^+^—H⋯O^−^ hydrogen bonds, which form one-dimensional hydrogen-bonded ladders. Salts (II), (III), (IV), (V) and (VII) have a type **II** ladder system despite the presence of halogen bonding and other inter­molecular inter­actions, whereas (VI) has a type **III** ladder system. Salt (I) has a unique hydrogen-bonded system of ladders, featuring both N^+^—H⋯O^−^ and N^+^—H⋯N hydrogen bonds owing to the presence of the pyridine functional group. The presence of an additional hydrogen-bond acceptor on the carboxyl­ate cation disrupts the formation of the ubiquitous type **II** and **III** ladder found predominately in ammonium carboxyl­ate salts. Halogen bonding, however, has no influence on their formation.

## Abstract 2

Hydrogen-bonded ladders typically encountered in ammonium carboxyl­ate salts did not form in the presence of a pyridine acceptor group.

## Chemical context   

Crystal engineering, the conception and synthesis of mol­ecular solid-state structures, is fundamentally based upon the discernment and subsequent exploitation of inter­molecular inter­actions. Thus, primarily non-covalent bonding is used to achieve the organization of mol­ecules and ions in the solid state in order to produce materials with desired properties. Examples of such materials include organic field-effect trans­istors, hole collectors in organic photovoltaic cells (Snaith, 2013 ▸), laser materials (Tessler, 1999 ▸) as well as organic light-emitting diodes and semiconductors (Odom *et al.*, 2003 ▸). The two principle forces exploited in the design of mol­ecular solids are hydrogen bonding and coordination complexation (Desiraju, 1989 ▸).

This work will focus on the effects of hydrogen bonding. In particular, we have investigated the effects thereof of changing both the structure and stereochemistry of the constituents on the robust ionic supra­molecular heterosynthons generated by ammonium carboxyl­ate salts (*R*–NH_3_^+^)·(*R*–COO^−^), where *R* often contains a phenyl­ethyl group generating chiral mol­ecules (Kinbara *et al.*, 1996 ▸). It is known from a wide variety of structural studies that ammonium carboxyl­ate salts predom­inately form two types of hydrogen-bonded one-dimensional ladders in the solid state (Odendal *et al.*, 2010 ▸). These are classified as type **II** and type **III**, where type **II** consists of repeating hydrogen-bonded rings with the descriptor 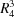
(10) (Bernstein *et al.*, 1995 ▸), while type **III** consists of alternating 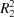
(8) and 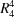
(12) rings. The robustness and perturbation of these ladders as a function of the structure and stereochemistry of the constituent ions have been tested *via* the crystallization of a variety of ammonium carboxyl­ate salts. The seven salts reported here are (see Scheme): (*RS*)-1-phenyl­ethan-1-aminium isonicotinate, (I), (*RS*)-1-phenyl­ethan-1-aminium flurbiprofenate, (II), (*RS*)-1-phenyl­ethan-1-aminium 2-chloro-4-nitro-benzoate, (III), (*RS*)-1-phenyl­ethan-1-aminium 4-iodo­benzoate, (IV), (*S*)-1-cyclo­hexyl­ethan-1-aminium 2-chloro-4-nitro-benzoate, (V), 2-(cyclo­hex-1-en-1-yl)ethan-1-aminium 4-bromo­benzoate, (VI), and (*S*)-1-cyclo­hexyl­ethan-1-aminium 4-bromo­benzoate, (VII).
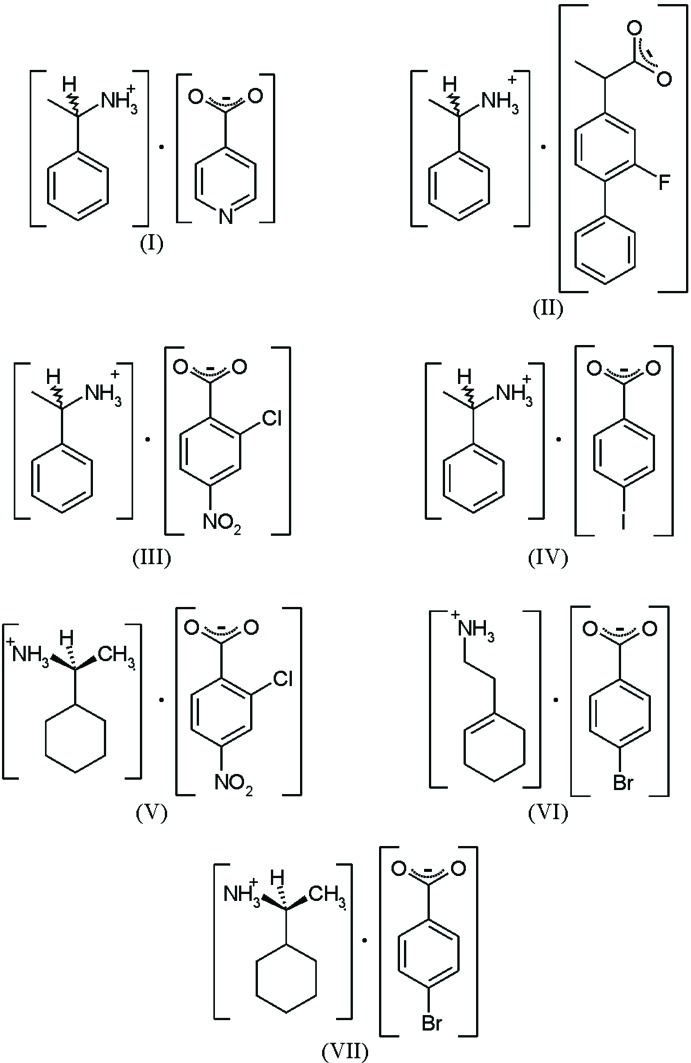


## Structural commentary   

**Figure 1:**
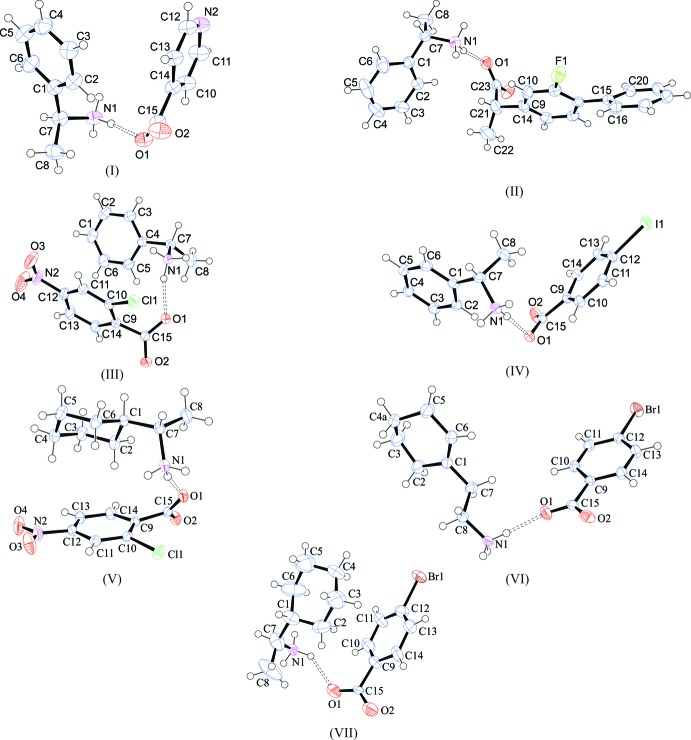
Perspective views of compounds (I)–(VII), showing the atom-numbering schemes. Displacement ellipsoids are drawn at the 50% probability level and H atoms are shown as small spheres of arbitrary radii. The dashed lines indicate the symmetry-independent N^+^—H⋯O^−^ or N^+^—H⋯N hydrogen bonds.

An amine and a carb­oxy­lic acid will combine to form a salt if the difference in p*K*_a_’s is approximately 3 or greater (Bhogala *et al.*, 2005 ▸; Lemmerer *et al.*, 2015 ▸). Thus, from the differences in p*K*_a_ values depicted in Table S1 in the supporting information, all the compounds considered in this work should be in the form of salts and hence possess charge-assisted hydrogen bonds, which are considered to be a stronger and more robust supra­molecular synthon than the same between neutral mol­ecules (Lemmerer *et al.*, 2008*a* ▸). All structures crystallize with a 1:1 ratio of ammonium cation to benzoate anion, with all mol­ecules on general positions. The asymmetric units and atom-numbering schemes are shown in Fig. 1 ▸.

## Supra­molecular features   

**Figure 2:**
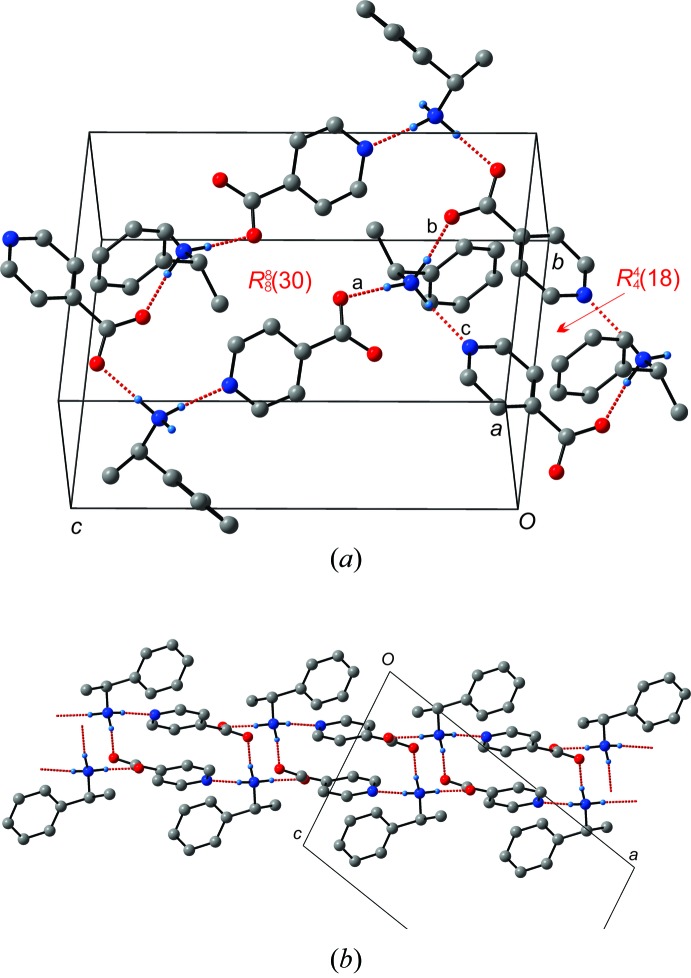
(*a*) Detailed view of the three hydrogen bonds forming two types of hydrogen-bonded rings in (I). (*b*) Side-on view of the two-dimensional, hydrogen-bonded layers formed.

Salt (I) consists of one 1-phenyl­ethan-1-aminium cation and one isonicotinate anion. The ammonium group forms three charge-assisted hydrogen bonds, shown in Fig. 2 ▸ *a*. The first of these bonds involves the O2 atom of the isonicotinate anion (i) (see Table 1 ▸) and is designated *a*. The second involves the O1 atom of the isonicotinate anion in the asymmetric unit and is designated *b*. The third involves the pyridine ring nitro­gen of a third isonicotinate anion (ii) and is designated *c*. The *b* and *c* hydrogen bonds form a ring structure involving two of each kind of bond, consisting of two mol­ecules of both 1-phenyl­ethan-1-aminium and isonicotinate (See Fig. 2 ▸ *a*). The graph set of this pattern is 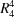
(18). A larger R^8^_8_(30) ring is formed using all three hydrogen bonds involving four of both 1-phenyl­ethan-1-aminium and isonicotinate ions. Overall, this forms a 2-D sheet as shown in Fig. 2 ▸ *b*. As neither of the two expected type **II** or type **III** ladders are formed it seems that the additional hydrogen-bond acceptor in the form of the nitro­gen atom of the pyridine ring disrupts their formation.

**Figure 3:**
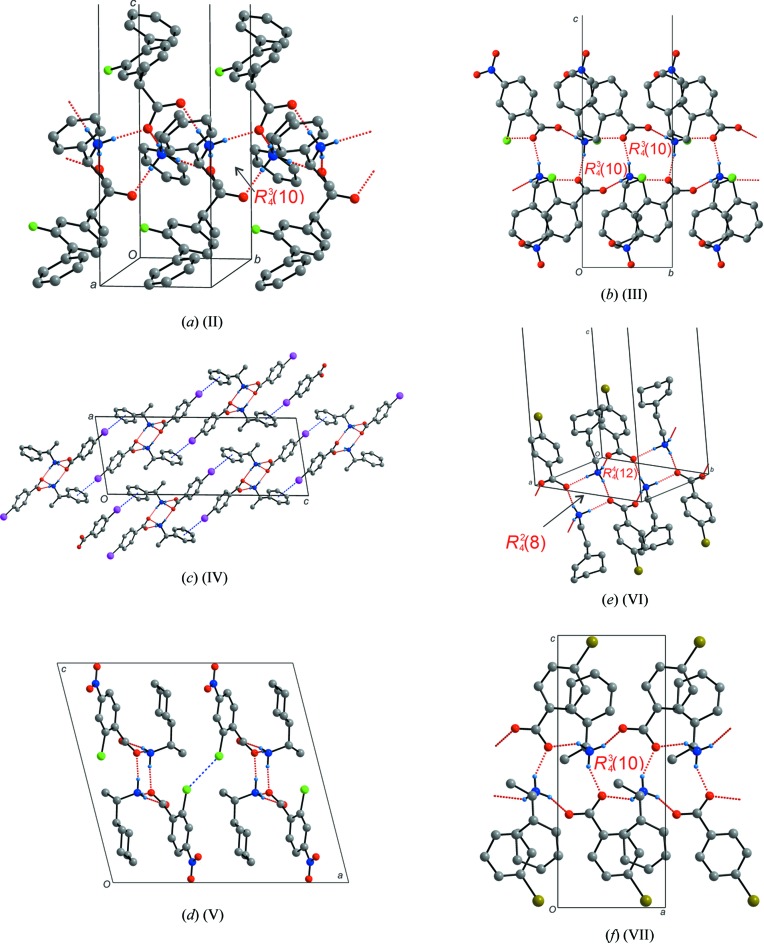
The hydrogen bonding (shown as dashed red lines), halogen bonding (shown as dashed blue lines) and packing diagrams for salts (II)–(VII).

In salt (II), the asymmetric unit consists of one 1-phenyl­ethan-1-aminium cation and one flurbiprofenate anion. Once again the ammonium group of the 1-phenyl­ethan-1-aminium ion forms three charged-assisted hydrogen bonds (Table 2 ▸). The first of these bonds involves the O2 atom of the anion while the other two involve the O1 atoms of the carboxyl­ate group of two separate symmetry-related flurbiprofenate anions. These three hydrogen bonds form a type **II** ladder system where each of the O1 atoms behaves as a bifurcated hydrogen-bond acceptor, linking the rings (Fig. 3 ▸ *a*). This pattern has translational symmetry through a twofold screw axis along the crystallographic *b* axis which is inherent in the space group *P*2_1_/*n*. As no short contacts such as halogen bonding or π-halogen inter­actions are observed, the fluorine atom does not disrupt the formation of the expected hydrogen-bonding patterns. However a peculiarity exists. As the cation was present as a racemate, traditionally type **III** ladders are expected to dominate as reported by Lemmerer and co-workers (Lemmerer *et al.*, 2008*b* ▸).

In salt (III), the asymmetric unit consists of one 1-phenyl­ethan-1-aminium cation and one 2-chloro-4-nitro-benzoate anion. The ammonium ion forms three charge-assisted hydrogen bonds to the carboxyl­ate group and not to the nitro group of the 2-chloro-4-nitro-benzoate anion (Table 3 ▸). In fact, no relevant non-covalent inter­actions involving the nitro group are observed. As for compound (II), a type **II** ladder is formed by the above-mentioned hydrogen bonds, as shown in Fig. 3 ▸ *b*. The anions in adjacent rings (related by translation along the *b* axis) are connected *via* C—O⋯Cl halogen bonds [O⋯Cl = 3.225 (1) Å; C—O⋯Cl = 160.5 (1)°]. However, this inter­action does not perturb the ladder supra­molecular synthons to a significant enough degree to prevent their formation. Once again, both enanti­omers of the 1-phenyl­ethan-1-aminium were present and thus type **III** ladders were expected to form.

In salt (IV), the asymmetric unit consists of one α-methyl-benzyl­ammonium cation and one 4-iodo­benzoate anion. A type **II** ladder system is observed (Table 4 ▸). An inter­esting feature of this structure is the π⋯halogen inter­action between the centre of the aromatic ring of the methyl­benzyl­ammonium cation and the iodine atom (Fig. 3 ▸ *c*). This is possible as, due to its size, iodine is very polarizable and thus the delocalized electrons in the aromatic system can create a permanent dipole in the iodine atom in the solid state. The distance of 3.740 (3) Å is similar to other mol­ecules containing iodine inter­acting non-covalently with aromatic systems reported in the literature (Nagels *et al.*, 2013 ▸). Again, as in salt (III), the halogen bonding does not disrupt the formation of the ladder motif.

In salt (V), the asymmetric unit consists of one (*S*)-1-cyclo­hexyl­ethyl­ammonium cation and one 2-chloro-4-nitro-benzoate anion, both on general positions. A type **II** ladder is formed as shown in Fig. 3 ▸ *d*. No hydrogen bonding to the nitro group takes place (Table 5 ▸), which is consistent with the results for salt (III). However, a type I Cl⋯Cl halogen bond is observed with a distance of 3.207 (3) Å that connects adjacent ladders along the *a* axis. As the cation is present as a single enanti­omer, the type **II** ladder formation is in line with the previous studies.

In salt (VI), the asymmetric unit consists of one 2-(1-cyclo­hexen­yl)ethyl­ammonium cation and one 4-bromo­benzoate anion, both on general positions. A type **III** ladder is observed (Table 6 ▸), unique among the salts here reported (Fig. 3 ▸ *e*). As in salt (V), the crystal structure is stabilized by halogen bonding, in this case between bromine and oxygen O1 with a distance of 3.253 (3) Å. The halogen bond connects adjacent ladders related by the two-fold screw axis.

In salt (VII), the asymmetric unit consists of one (*S*)-1-cyclo­hexyl­ethyl­ammonium cation and one 4-bromo­benzoate anion, both on general positions. A type **II** ladder is formed (Table 7 ▸, Fig. 3 ▸ *f*). This is expected as the cation is enanti­omerically pure (Lemmerer *et al.*, 2008*b* ▸). In contrast to the previous salts that have a halogen atom on the anion, no halogen bonding is observed.

In summary, introducing a pyridine functional group instead of a plain benzene in (I) has altered the hydrogen-bonding pattern usually observed in ammonium carboxyl­ate salts, which generally show the typical type **II** and **III** patterns as seen in (II)–(VII)

## Synthesis and crystallization   

All chemicals were purchased from commercial sources (Sigma Aldrich) and used as received without further purification. Crystals were grown *via* the slow evaporation of methanol or ethanol solutions under ambient conditions. All solutions contained a 1:1 ratio of amine and acid. Detailed masses and volumes are as follows. For (I): (*RS*)-α-methyl­benzyl­amine (0.100 g, 0.825 mmol) and isonicotinic acid (0.102 g, 0.825 mmol) in methanol (5 mL); for (II): (*RS*)-α-methyl­benzyl­amine (0.100 g, 0.825 mmol) and flurbiprofen (0.202 g, 0.825 mmol) in ethanol (8 mL); for (III): (*RS*)-α-methyl­benzyl­amine (0.100 g, 0.825 mmol) and 2-chloro-4-nitro-benzoic acid (0.166 g, 0.825 mmol) in ethanol (5 mL); for (IV): (*RS*)-α-methyl­benzyl­amine (0.492 g, 0.406 mmol) and 4-iodo­benzoic acid (0.101 g, 0.406 mmol) in ethanol (5 mL); for (V): (*S*)-1-cyclo­hexyl­ethyl­amine (0.0254 g, 0.200 mmol) and 2-chloro-4-nitro-benzoic acid (0.0403 g, 0.200 mmol); for (VI): 2-(1-cyclo­hexen­yl) ethyl­amine (0.0250 g, 0.200 mmol) and 4-bromo­benzoic acid (0.0410 g, 0.200 mmol); and for (VII): (*S*)-1-cyclo­hexyl­ethyl­amine (0.0254 g, 0.200 mmol) and 4-bromo­benzoic acid (0.0410 g, 0.200 mmol).

## Refinement details   

Crystal data, data collection and structure refinement details are summarized in Table 8 ▸. For all compounds, the C-bound H atoms were placed geometrically [C—H bond lengths of 1.00 Å (methine CH), 0.99 Å (ethyl­ene CH_2_), 0.98 Å (methyl­ene CH_3_) and 0.95 Å (Ar—H)] and refined as riding with *U*_iso_(H) = 1.2*U*_eq_(C) or *U*_iso_(H) = 1.5*U*_eq_(C). The N-bound H atoms were located in difference-Fourier maps and their coordinates and isotropic displacement parameters allowed to refine freely for (I)–(VI). For (VII), the N-bound H atoms were geometrically placed (C—H bond lengths of 0.91 Å) and refined as riding with *U*_iso_(H) = 1.5*U*_eq_(C).

For the disorder of the atom C4 in the cyclo­hexene ring of (VI), two alternate positions were found in a difference-Fourier map, and their occupancies refined to final values of 0.77 (2) and 0.23 (2).

## Related literature   

The following references, not cited in the main body of the paper, have been cited in the supporting information: Bouchard *et al.* (2002 ▸); Isoda *et al.* (1997 ▸); Perrin (1982 ▸); Portnov *et al.* (1971 ▸); van Sorge *et al.* (1999 ▸); Tuckerman *et al.* (1959 ▸).

## Supplementary Material

Crystal structure: contains datablock(s) I, II, III, IV, V, VI, VII, shelx. DOI: 10.1107/S2056989017017856/xi2001sup1.cif

Structure factors: contains datablock(s) I. DOI: 10.1107/S2056989017017856/xi2001Isup2.hkl

Structure factors: contains datablock(s) II. DOI: 10.1107/S2056989017017856/xi2001IIsup3.hkl

Structure factors: contains datablock(s) III. DOI: 10.1107/S2056989017017856/xi2001IIIsup4.hkl

Structure factors: contains datablock(s) IV. DOI: 10.1107/S2056989017017856/xi2001IVsup5.hkl

Structure factors: contains datablock(s) V. DOI: 10.1107/S2056989017017856/xi2001Vsup6.hkl

Structure factors: contains datablock(s) VI. DOI: 10.1107/S2056989017017856/xi2001VIsup7.hkl

Structure factors: contains datablock(s) VII. DOI: 10.1107/S2056989017017856/xi2001VIIsup8.hkl

Supporting information file. DOI: 10.1107/S2056989017017856/xi2001Isup9.cml

Supporting information file. DOI: 10.1107/S2056989017017856/xi2001IIsup10.cml

Supporting information file. DOI: 10.1107/S2056989017017856/xi2001IIIsup11.cml

Supporting information file. DOI: 10.1107/S2056989017017856/xi2001IVsup12.cml

Supporting information file. DOI: 10.1107/S2056989017017856/xi2001Vsup13.cml

Supporting information file. DOI: 10.1107/S2056989017017856/xi2001VIsup14.cml

CCDC references: 1811019, 1811018, 1811017, 1811016, 1811015, 1811014, 1811013

Additional supporting information:  crystallographic information; 3D view; checkCIF report

## Figures and Tables

**Table 1:** Hydrogen-bond geometry (Å, °) for (I)

*D*—H⋯*A*	*D*—H	H⋯*A*	*D*⋯*A*	*D*—H⋯*A*
N1—H1*B*⋯O2^i^	0.95 (2)	1.80 (2)	2.747 (2)	176 (2)
N1—H1*C*⋯O1	0.98 (2)	1.81 (2)	2.783 (2)	174 (2)
N1—H1*A*⋯N2^ii^	0.93 (2)	1.93 (2)	2.856 (2)	175 (2)

Symmetry codes: (i) 
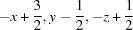
; (ii) 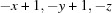.

**Table 2:** Hydrogen-bond geometry (Å, °) for (II)

*D*—H⋯*A*	*D*—H	H⋯*A*	*D*⋯*A*	*D*—H⋯*A*
N1—H1*A*⋯O1^i^	0.91 (3)	1.91 (3)	2.809 (3)	170 (3)
N1—H1*C*⋯O1^ii^	0.90 (3)	1.87 (3)	2.758 (3)	168 (3)
N1—H1*B*⋯O2	0.95 (3)	1.75 (3)	2.693 (3)	176 (3)

Symmetry codes: (i) 
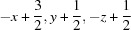
; (ii) 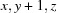.

**Table 3:** Hydrogen-bond geometry (Å, °) for (III)

*D*—H⋯*A*	*D*—H	H⋯*A*	*D*⋯*A*	*D*—H⋯*A*
N1—H1*A*⋯O1	0.94 (2)	1.91 (2)	2.829 (1)	165 (1)
N1—H1*B*⋯O2^i^	0.94 (2)	1.85 (2)	2.789 (1)	176 (1)
N1—H1*C*⋯O1^ii^	0.95 (2)	1.84 (2)	2.780 (1)	170 (1)

Symmetry codes: (i) 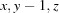; (ii) 
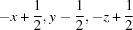
.

**Table 4:** Hydrogen-bond geometry (Å, °) for (IV)

*D*—H⋯*A*	*D*—H	H⋯*A*	*D*⋯*A*	*D*—H⋯*A*
N1—H1*A*⋯O1	0.88 (3)	1.92 (3)	2.796 (3)	175 (2)
N1—H1*B*⋯O2^i^	0.84 (3)	1.88 (3)	2.715 (3)	174 (3)
N1—H1*C*⋯O1^ii^	0.92 (3)	1.83 (3)	2.735 (2)	169 (3)

Symmetry codes: (i) 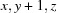; (ii) 
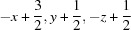
.

**Table 5:** Hydrogen-bond geometry (Å, °) for (V)

*D*—H⋯*A*	*D*—H	H⋯*A*	*D*⋯*A*	*D*—H⋯*A*
N1—H1*A*⋯O1	0.93 (3)	1.96 (3)	2.869 (2)	167 (2)
N1—H1*B*⋯O1^i^	0.93 (3)	1.86 (3)	2.785 (2)	173 (2)
N1—H1*C*⋯O2^ii^	0.88 (3)	1.99 (3)	2.858 (2)	170 (2)

Symmetry codes: (i) 
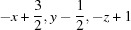
; (ii) 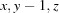.

**Table 6:** Hydrogen-bond geometry (Å, °) for (VI)

*D*—H⋯*A*	*D*—H	H⋯*A*	*D*⋯*A*	*D*—H⋯*A*
N1—H1*A*⋯O1	0.86 (3)	1.89 (3)	2.737 (2)	167 (3)
N1—H1*B*⋯O2^i^	0.94 (3)	1.85 (3)	2.763 (3)	164 (3)
N1—H1*C*⋯O2^ii^	0.86 (3)	1.89 (3)	2.727 (2)	167 (3)

Symmetry codes: (i) 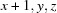; (ii) 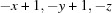.

**Table 7:** Hydrogen-bond geometry (Å, °) for (VII)

*D*—H⋯*A*	*D*—H	H⋯*A*	*D*⋯*A*	*D*—H⋯*A*
N1—H1*A*⋯O1	0.91	1.99	2.781 (12)	144
N1—H1*B*⋯O2^i^	0.91	2.06	2.870 (12)	148
N1—H1*C*⋯O1^ii^	0.91	1.89	2.718 (10)	150

Symmetry codes: (i) 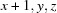; (ii) 
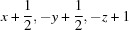
.

**Table:** 

	(I)	(II)	(III)	(IV)
Crystal data				
Chemical formula	C_8_H_12_N^+^·C_6_H_4_NO_2_^−^	C_8_H_12_N^+^·C_15_H_12_FO_2_^−^	C_8_H_12_N^+^·C_7_H_3_ClNO_4_^−^	C_8_H_12_N^+^·C_7_H_4_IO_2_^−^
*M*_r_	244.29	365.43	322.74	369.19
Crystal system, space group	Monoclinic, *P*2_1_/*n*	Monoclinic, *P*2_1_/*n*	Monoclinic, *C*2/*c*	Monoclinic, *P*2_1_/*n*
Temperature (K)	173	173	173	173
*a*, *b*, *c* (Å)	9.4094 (5), 9.4697 (5), 15.1613 (9)	12.4146 (4), 5.9101 (2), 27.3645 (9)	15.5817 (7), 6.3914 (3), 31.3238 (14)	9.7224 (5), 6.0571 (3), 24.8767 (12)
α, β, γ (°)	90, 102.247 (3), 90	90, 90.793 (1), 90	90, 100.998 (2), 90	90, 99.527 (2), 90
*V* (Å^3^)	1320.19 (13)	2007.58 (11)	3062.2 (2)	1444.77 (12)
*Z*	4	4	8	4
Radiation type	Mo *K*α	Mo *K*α	Mo *K*α	Mo *K*α
μ (mm^−1^)	0.08	0.08	0.27	2.21
Crystal size (mm)	0.76 × 0.33 × 0.07	0.49 × 0.05 × 0.03	0.47 × 0.35 × 0.08	0.68 × 0.16 × 0.04

Data collection				
Diffractometer	Bruker D8 Venture Photon CCD area detector	Bruker D8 Venture Photon CCD area detector	Bruker D8 Venture Photon CCD area detector	Bruker D8 Venture Photon CCD area detector
Absorption correction	Integration (*XPREP*; Bruker, 2016 ▸)	Integration (*XPREP*; Bruker, 2016 ▸)	Integration (*XPREP*; Bruker, 2016 ▸)	Integration (*XPREP*; Bruker, 2016 ▸)
*T*_min_, *T*_max_	0.95, 0.96	0.984, 0.998	0.903, 0.979	0.508, 0.928
No. of measured, independent and observed [*I* > 2σ(*I*)] reflections	38822, 3190, 2359	21119, 3729, 3014	18885, 3709, 3218	30978, 3463, 3109
*R*_int_	0.101	0.036	0.053	0.051

Refinement				
*R*[*F* ^2^ > 2σ(*F* ^2^)], *wR*(*F* ^2^), *S*	0.056, 0.171, 1.05	0.061, 0.174, 1.07	0.035, 0.091, 1.04	0.026, 0.057, 1.13
No. of reflections	3190	3729	3709	3463
No. of parameters	176	256	212	185
No. of restraints	0	0	0	0
H-atom treatment	H atoms treated by a mixture of independent and constrained refinement	H atoms treated by a mixture of independent and constrained refinement	H atoms treated by a mixture of independent and constrained refinement	H atoms treated by a mixture of independent and constrained refinement
Δρ_max_, Δρ_min_ (e Å^−3^)	0.38, −0.40	1.00, −0.30	0.28, −0.32	0.74, −0.38

**Table:** 

	(V)	(VI)	(VII)
Crystal data			
Chemical formula	C_8_H_18_N^+^·C_7_H_3_ClNO_4_^−^	C_8_H_15_N^+^·C_7_H_4_BrO_2_^−^	C_8_H_18_N^+^·C_7_H_4_BrO_2_^−^
*M*_r_	328.79	325.22	328.24
Crystal system, space group	Monoclinic, *C*2	Monoclinic, *P*2_1_/*n*	Orthorhombic, *P*2_1_2_1_2_1_
Temperature (K)	173	173	173
*a*, *b*, *c* (Å)	16.2280 (15), 6.4392 (5), 15.5937 (15)	6.4391 (3), 17.0023 (8), 14.1588 (6)	6.2790 (3), 15.6610 (9), 15.8800 (8)
α, β, γ (°)	90, 104.289 (4), 90	90, 102.241 (2), 90	90, 90, 90
*V* (Å^3^)	1579.1 (2)	1514.86 (12)	1561.57 (14)
*Z*	4	4	4
Radiation type	Mo *K*α	Mo *K*α	Mo *K*α
μ (mm^−1^)	0.26	2.71	2.63
Crystal size (mm)	0.51 × 0.39 × 0.06	0.68 × 0.18 × 0.1	0.69 × 0.13 × 0.10

Data collection			
Diffractometer	Bruker D8 Venture Photon CCD area detector	Bruker D8 Venture Photon CCD area detector	Bruker D8 Venture Photon CCD area detector
Absorption correction	Integration (*XPREP*; Bruker, 2016 ▸)	Integration (*XPREP*; Bruker, 2016 ▸)	Integration (*XPREP*; Bruker, 2016 ▸)
*T*_min_, *T*_max_	0.910, 0.988	0.275, 0.776	0.452, 0.846
No. of measured, independent and observed [*I* > 2σ(*I*)] reflections	15055, 3837, 3587	30862, 3658, 3302	21006, 2913, 2687
*R*_int_	0.045	0.071	0.068

Refinement			
*R*[*F* ^2^ > 2σ(*F* ^2^)], *wR*(*F* ^2^), *S*	0.032, 0.071, 1.04	0.037, 0.098, 1.05	0.078, 0.211, 1.08
No. of reflections	3837	3658	2913
No. of parameters	211	194	174
No. of restraints	1	0	0
H-atom treatment	H atoms treated by a mixture of independent and constrained refinement	H atoms treated by a mixture of independent and constrained refinement	H-atom parameters constrained
Δρ_max_, Δρ_min_ (e Å^−3^)	0.20, −0.18	1.01, −1.02	1.46, −0.48
Absolute structure	Flack *x* determined using 1512 quotients [(*I* ^+^)−(*I* ^−^)]/[(*I* ^+^)+(*I* ^−^)] (Parsons *et al.*, 2013 ▸).	–	Flack *x* determined using 1026 quotients [(*I* ^+^)−(*I* ^−^)]/[(*I* ^+^)+(*I* ^−^)] (Parsons *et al.*, 2013 ▸)
Absolute structure parameter	−0.031 (19)	–	0.068 (9)

Computer programs: *APEX3*, *SAINT-Plus* and *XPREP* (Bruker, 2016 ▸), *SHELXS97* (Sheldrick, 2008 ▸), *SHELXL2017* (Sheldrick, 2015 ▸), *ORTEP-3 for Windows* and *WinGX* (Farrugia, 2012 ▸) and *DIAMOND* (Brandenburg & Berndt, 1999 ▸).

## supplementary crystallographic information

### (RS)-1-Phenylethan-1-aminium pyridine-4-carboxylate (I) Crystal data

**Table:**

C_8_H_12_N^+^·C_6_H_4_NO_2_^−^	*F*(000) = 520
*M**_r_* = 244.29	*D*_x_ = 1.229 Mg m^−^^3^
Monoclinic, *P*2_1_/*n*	Mo *K*α radiation, λ = 0.71073 Å
Hall symbol: -P 2yn	Cell parameters from 8190 reflections
*a* = 9.4094 (5) Å	θ = 2.4–24.1°
*b* = 9.4697 (5) Å	µ = 0.08 mm^−^^1^
*c* = 15.1613 (9) Å	*T* = 173 K
β = 102.247 (3)°	Plate, colourless
*V* = 1320.19 (13) Å^3^	0.76 × 0.33 × 0.07 mm
*Z* = 4	

### (RS)-1-Phenylethan-1-aminium pyridine-4-carboxylate (I) Data collection

**Table:**

Bruker D8 Venture Photon CCD area detector diffractometer	2359 reflections with *I* > 2σ(*I*)
Graphite monochromator	*R*_int_ = 0.101
ω scans	θ_max_ = 28.0°, θ_min_ = 2.4°
Absorption correction: integration (XPREP; Bruker, 2016)	*h* = −12→12
*T*_min_ = 0.95, *T*_max_ = 0.96	*k* = −12→12
38822 measured reflections	*l* = −20→20
3190 independent reflections	

### (RS)-1-Phenylethan-1-aminium pyridine-4-carboxylate (I) Refinement

**Table:**

Refinement on *F*^2^	0 constraints
Least-squares matrix: full	Hydrogen site location: mixed
*R*[*F*^2^ > 2σ(*F*^2^)] = 0.056	H atoms treated by a mixture of independent and constrained refinement
*wR*(*F*^2^) = 0.171	*w* = 1/[σ^2^(*F*_o_^2^) + (0.0941*P*)^2^ + 0.2604*P*] where *P* = (*F*_o_^2^ + 2*F*_c_^2^)/3
*S* = 1.05	(Δ/σ)_max_ < 0.001
3190 reflections	Δρ_max_ = 0.38 e Å^−^^3^
176 parameters	Δρ_min_ = −0.40 e Å^−^^3^
0 restraints	

### (RS)-1-Phenylethan-1-aminium pyridine-4-carboxylate (I) Special details

**Table:**

Experimental. Numerical integration absorption corrections based on indexed crystal faces were applied using the XPREP routine (Bruker, 2016)
Geometry. All esds (except the esd in the dihedral angle between two l.s. planes) are estimated using the full covariance matrix. The cell esds are taken into account individually in the estimation of esds in distances, angles and torsion angles; correlations between esds in cell parameters are only used when they are defined by crystal symmetry. An approximate (isotropic) treatment of cell esds is used for estimating esds involving l.s. planes.

### (RS)-1-Phenylethan-1-aminium pyridine-4-carboxylate (I) Fractional atomic coordinates and isotropic or equivalent isotropic displacement parameters (Å^2^)

**Table:**

	*x*	*y*	*z*	*U*_iso_*/*U*_eq_	
C1	0.36397 (17)	0.73101 (15)	0.23522 (11)	0.0356 (4)	
C2	0.37700 (18)	0.82392 (16)	0.16665 (12)	0.0379 (4)	
H2	0.469713	0.861634	0.164378	0.045*	
C3	0.25688 (19)	0.86235 (18)	0.10161 (13)	0.0427 (4)	
H3	0.267388	0.924437	0.054193	0.051*	
C4	0.1213 (2)	0.8100 (2)	0.10587 (13)	0.0475 (4)	
H4	0.038141	0.837452	0.06192	0.057*	
C5	0.1068 (2)	0.7180 (2)	0.17387 (15)	0.0536 (5)	
H5	0.013626	0.68193	0.176604	0.064*	
C6	0.22735 (19)	0.6783 (2)	0.23802 (14)	0.0471 (4)	
H6	0.216714	0.614276	0.284453	0.057*	
C7	0.49410 (18)	0.68555 (16)	0.30626 (11)	0.0368 (4)	
H7	0.45852	0.621973	0.349619	0.044*	
C8	0.5745 (2)	0.80785 (18)	0.35973 (13)	0.0482 (5)	
H8A	0.657613	0.771497	0.404167	0.072*	
H8B	0.508569	0.858155	0.390937	0.072*	
H8C	0.609192	0.872738	0.318543	0.072*	
C10	0.71054 (19)	0.61304 (16)	0.01922 (13)	0.0421 (4)	
H10	0.783407	0.575032	0.066354	0.051*	
C11	0.6532 (2)	0.53314 (17)	−0.05587 (13)	0.0475 (5)	
H11	0.690245	0.440511	−0.059585	0.057*	
C12	0.49964 (18)	0.71011 (17)	−0.11664 (12)	0.0389 (4)	
H12	0.424163	0.744187	−0.163695	0.047*	
C13	0.55272 (17)	0.79860 (15)	−0.04510 (11)	0.0363 (4)	
H13	0.515779	0.891799	−0.044027	0.044*	
C14	0.66020 (16)	0.75029 (15)	0.02501 (11)	0.0320 (3)	
C15	0.72087 (15)	0.84180 (15)	0.10609 (11)	0.0312 (3)	
N1	0.59829 (15)	0.60289 (13)	0.26409 (10)	0.0340 (3)	
N2	0.54883 (16)	0.57833 (14)	−0.12346 (10)	0.0420 (4)	
O1	0.76355 (12)	0.78218 (11)	0.18042 (8)	0.0382 (3)	
O2	0.72283 (14)	0.97236 (11)	0.09234 (8)	0.0446 (3)	
H1A	0.549 (2)	0.541 (2)	0.2211 (14)	0.047 (5)*	
H1B	0.664 (2)	0.560 (2)	0.3127 (15)	0.052 (6)*	
H1C	0.651 (2)	0.666 (2)	0.2308 (13)	0.048 (5)*	

### (RS)-1-Phenylethan-1-aminium pyridine-4-carboxylate (I) Atomic displacement parameters (Å^2^)

**Table:**

	*U*^11^	*U*^22^	*U*^33^	*U*^12^	*U*^13^	*U*^23^
C1	0.0372 (8)	0.0231 (7)	0.0463 (9)	0.0016 (6)	0.0086 (7)	−0.0025 (6)
C2	0.0364 (8)	0.0255 (7)	0.0519 (10)	−0.0001 (6)	0.0096 (7)	0.0012 (6)
C3	0.0484 (10)	0.0293 (8)	0.0496 (10)	0.0068 (7)	0.0083 (8)	0.0036 (7)
C4	0.0390 (9)	0.0436 (10)	0.0559 (11)	0.0110 (7)	0.0014 (8)	−0.0045 (8)
C5	0.0341 (9)	0.0560 (12)	0.0707 (13)	−0.0025 (8)	0.0108 (9)	0.0024 (10)
C6	0.0417 (10)	0.0423 (9)	0.0589 (11)	−0.0042 (7)	0.0140 (8)	0.0066 (8)
C7	0.0404 (9)	0.0261 (7)	0.0431 (9)	0.0004 (6)	0.0069 (7)	0.0009 (6)
C8	0.0546 (11)	0.0343 (9)	0.0517 (11)	0.0016 (7)	0.0022 (8)	−0.0112 (7)
C10	0.0445 (9)	0.0232 (7)	0.0519 (10)	0.0068 (6)	−0.0048 (7)	−0.0022 (7)
C11	0.0547 (10)	0.0228 (8)	0.0590 (11)	0.0073 (7)	−0.0015 (9)	−0.0078 (7)
C12	0.0375 (8)	0.0281 (8)	0.0467 (9)	0.0012 (6)	−0.0008 (7)	−0.0020 (6)
C13	0.0375 (8)	0.0208 (7)	0.0477 (9)	0.0031 (6)	0.0024 (7)	−0.0002 (6)
C14	0.0313 (7)	0.0194 (6)	0.0442 (8)	−0.0012 (5)	0.0055 (6)	0.0002 (6)
C15	0.0279 (7)	0.0211 (7)	0.0429 (8)	0.0007 (5)	0.0033 (6)	0.0001 (6)
N1	0.0360 (7)	0.0182 (6)	0.0443 (8)	0.0009 (5)	0.0006 (6)	0.0011 (5)
N2	0.0465 (8)	0.0250 (7)	0.0510 (8)	−0.0025 (6)	0.0026 (6)	−0.0068 (6)
O1	0.0390 (6)	0.0294 (6)	0.0423 (7)	−0.0036 (4)	0.0000 (5)	0.0034 (5)
O2	0.0582 (8)	0.0181 (5)	0.0509 (7)	−0.0017 (5)	−0.0036 (6)	−0.0010 (5)

### (RS)-1-Phenylethan-1-aminium pyridine-4-carboxylate (I) Geometric parameters (Å, º)

**Table:**

C1—C2	1.387 (2)	C8—H8C	0.98
C1—C6	1.388 (2)	C10—C11	1.378 (2)
C1—C7	1.512 (2)	C10—C14	1.392 (2)
C2—C3	1.382 (2)	C10—H10	0.95
C2—H2	0.95	C11—N2	1.331 (2)
C3—C4	1.383 (3)	C11—H11	0.95
C3—H3	0.95	C12—N2	1.343 (2)
C4—C5	1.378 (3)	C12—C13	1.378 (2)
C4—H4	0.95	C12—H12	0.95
C5—C6	1.381 (3)	C13—C14	1.381 (2)
C5—H5	0.95	C13—H13	0.95
C6—H6	0.95	C14—C15	1.513 (2)
C7—N1	1.500 (2)	C15—O1	1.2482 (18)
C7—C8	1.520 (2)	C15—O2	1.2546 (18)
C7—H7	1	N1—H1A	0.93 (2)
C8—H8A	0.98	N1—H1B	0.95 (2)
C8—H8B	0.98	N1—H1C	0.98 (2)
			
C2—C1—C6	118.74 (16)	H8A—C8—H8C	109.5
C2—C1—C7	121.84 (14)	H8B—C8—H8C	109.5
C6—C1—C7	119.41 (15)	C11—C10—C14	119.06 (16)
C3—C2—C1	120.86 (16)	C11—C10—H10	120.5
C3—C2—H2	119.6	C14—C10—H10	120.5
C1—C2—H2	119.6	N2—C11—C10	123.73 (15)
C2—C3—C4	119.69 (17)	N2—C11—H11	118.1
C2—C3—H3	120.2	C10—C11—H11	118.1
C4—C3—H3	120.2	N2—C12—C13	123.55 (16)
C5—C4—C3	120.01 (17)	N2—C12—H12	118.2
C5—C4—H4	120	C13—C12—H12	118.2
C3—C4—H4	120	C12—C13—C14	119.25 (14)
C4—C5—C6	120.16 (17)	C12—C13—H13	120.4
C4—C5—H5	119.9	C14—C13—H13	120.4
C6—C5—H5	119.9	C13—C14—C10	117.66 (15)
C5—C6—C1	120.53 (17)	C13—C14—C15	121.61 (13)
C5—C6—H6	119.7	C10—C14—C15	120.73 (14)
C1—C6—H6	119.7	O1—C15—O2	125.63 (15)
N1—C7—C1	110.42 (13)	O1—C15—C14	117.90 (13)
N1—C7—C8	109.18 (14)	O2—C15—C14	116.46 (14)
C1—C7—C8	113.54 (13)	C7—N1—H1A	110.8 (12)
N1—C7—H7	107.8	C7—N1—H1B	105.6 (12)
C1—C7—H7	107.8	H1A—N1—H1B	115.0 (18)
C8—C7—H7	107.8	C7—N1—H1C	110.6 (12)
C7—C8—H8A	109.5	H1A—N1—H1C	104.9 (16)
C7—C8—H8B	109.5	H1B—N1—H1C	110.0 (17)
H8A—C8—H8B	109.5	C11—N2—C12	116.73 (14)
C7—C8—H8C	109.5		
			
C6—C1—C2—C3	−0.7 (2)	C14—C10—C11—N2	−1.5 (3)
C7—C1—C2—C3	179.00 (15)	N2—C12—C13—C14	−1.4 (3)
C1—C2—C3—C4	1.4 (3)	C12—C13—C14—C10	0.4 (2)
C2—C3—C4—C5	−1.1 (3)	C12—C13—C14—C15	−178.95 (14)
C3—C4—C5—C6	0.2 (3)	C11—C10—C14—C13	0.9 (3)
C4—C5—C6—C1	0.5 (3)	C11—C10—C14—C15	−179.69 (16)
C2—C1—C6—C5	−0.2 (3)	C13—C14—C15—O1	148.63 (15)
C7—C1—C6—C5	−179.94 (17)	C10—C14—C15—O1	−30.7 (2)
C2—C1—C7—N1	−64.54 (19)	C13—C14—C15—O2	−30.9 (2)
C6—C1—C7—N1	115.18 (17)	C10—C14—C15—O2	149.70 (16)
C2—C1—C7—C8	58.5 (2)	C10—C11—N2—C12	0.6 (3)
C6—C1—C7—C8	−121.83 (18)	C13—C12—N2—C11	0.9 (3)

### (RS)-1-Phenylethan-1-aminium pyridine-4-carboxylate (I) Hydrogen-bond geometry (Å, º)

**Table:**

*D*—H···*A*	*D*—H	H···*A*	*D*···*A*	*D*—H···*A*
N1—H1*B*···O2^i^	0.95 (2)	1.80 (2)	2.747 (2)	176 (2)
N1—H1*C*···O1	0.98 (2)	1.81 (2)	2.783 (2)	174 (2)
N1—H1*A*···N2^ii^	0.93 (2)	1.93 (2)	2.856 (2)	175 (2)

Symmetry codes: (i) −*x*+3/2, *y*−1/2, −*z*+1/2; (ii) −*x*+1, −*y*+1, −*z*.

### (RS)-1-Phenylethan-1-aminium 2-(3-fluoro-4-phenylphenyl)propanoate (II) Crystal data

**Table:**

C_8_H_12_N^+^·C_15_H_12_FO_2_^−^	*F*(000) = 776
*M**_r_* = 365.43	*D*_x_ = 1.209 Mg m^−^^3^
Monoclinic, *P*2_1_/*n*	Mo *K*α radiation, λ = 0.71073 Å
Hall symbol: -P 2yn	Cell parameters from 8586 reflections
*a* = 12.4146 (4) Å	θ = 3.3–28.1°
*b* = 5.9101 (2) Å	µ = 0.08 mm^−^^1^
*c* = 27.3645 (9) Å	*T* = 173 K
β = 90.793 (1)°	Needle, colourless
*V* = 2007.58 (11) Å^3^	0.49 × 0.05 × 0.03 mm
*Z* = 4	

### (RS)-1-Phenylethan-1-aminium 2-(3-fluoro-4-phenylphenyl)propanoate (II) Data collection

**Table:**

Bruker D8 Venture Photon CCD area detector diffractometer	3014 reflections with *I* > 2σ(*I*)
Graphite monochromator	*R*_int_ = 0.036
ω scans	θ_max_ = 25.5°, θ_min_ = 3.0°
Absorption correction: integration (XPREP; Bruker, 2016)	*h* = −15→15
*T*_min_ = 0.984, *T*_max_ = 0.998	*k* = −7→7
21119 measured reflections	*l* = −33→33
3729 independent reflections	

### (RS)-1-Phenylethan-1-aminium 2-(3-fluoro-4-phenylphenyl)propanoate (II) Refinement

**Table:**

Refinement on *F*^2^	0 constraints
Least-squares matrix: full	Hydrogen site location: mixed
*R*[*F*^2^ > 2σ(*F*^2^)] = 0.061	H atoms treated by a mixture of independent and constrained refinement
*wR*(*F*^2^) = 0.174	*w* = 1/[σ^2^(*F*_o_^2^) + (0.0822*P*)^2^ + 1.6935*P*] where *P* = (*F*_o_^2^ + 2*F*_c_^2^)/3
*S* = 1.07	(Δ/σ)_max_ < 0.001
3729 reflections	Δρ_max_ = 1.00 e Å^−^^3^
256 parameters	Δρ_min_ = −0.29 e Å^−^^3^
0 restraints	

### (RS)-1-Phenylethan-1-aminium 2-(3-fluoro-4-phenylphenyl)propanoate (II) Special details

**Table:**

Experimental. Numerical integration absorption corrections based on indexed crystal faces were applied using the XPREP routine (Bruker, 2016)
Geometry. All esds (except the esd in the dihedral angle between two l.s. planes) are estimated using the full covariance matrix. The cell esds are taken into account individually in the estimation of esds in distances, angles and torsion angles; correlations between esds in cell parameters are only used when they are defined by crystal symmetry. An approximate (isotropic) treatment of cell esds is used for estimating esds involving l.s. planes.

### (RS)-1-Phenylethan-1-aminium 2-(3-fluoro-4-phenylphenyl)propanoate (II) Fractional atomic coordinates and isotropic or equivalent isotropic displacement parameters (Å^2^)

**Table:**

	*x*	*y*	*z*	*U*_iso_*/*U*_eq_	
C1	1.04935 (18)	0.8642 (4)	0.27025 (8)	0.0329 (5)	
C2	1.0760 (2)	1.0387 (5)	0.30184 (9)	0.0417 (6)	
H2	1.031358	1.168965	0.303281	0.05*	
C3	1.1675 (2)	1.0250 (6)	0.33148 (10)	0.0530 (7)	
H3	1.184766	1.1449	0.353363	0.064*	
C4	1.2332 (2)	0.8381 (6)	0.32915 (12)	0.0601 (8)	
H4	1.29587	0.828908	0.349338	0.072*	
C5	1.2079 (2)	0.6647 (6)	0.29759 (15)	0.0674 (9)	
H5	1.253609	0.536126	0.295793	0.081*	
C6	1.1162 (2)	0.6766 (5)	0.26840 (12)	0.0520 (7)	
H6	1.098886	0.555252	0.246917	0.062*	
C7	0.95219 (18)	0.8788 (4)	0.23622 (8)	0.0334 (5)	
H7	0.941499	0.727244	0.220658	0.04*	
C8	0.9665 (2)	1.0510 (5)	0.19606 (9)	0.0466 (7)	
H8A	1.031382	1.014686	0.177604	0.07*	
H8B	0.90358	1.047944	0.174065	0.07*	
H8C	0.973963	1.202164	0.210442	0.07*	
C9	0.72402 (18)	0.2856 (4)	0.39881 (8)	0.0358 (5)	
C10	0.66307 (19)	0.0970 (4)	0.38683 (8)	0.0367 (5)	
H10	0.694035	−0.023957	0.368967	0.044*	
C11	0.55715 (18)	0.0859 (4)	0.40101 (8)	0.0329 (5)	
C12	0.50615 (17)	0.2529 (4)	0.42747 (7)	0.0284 (5)	
C13	0.56906 (18)	0.4412 (4)	0.43937 (8)	0.0327 (5)	
H13	0.537912	0.561242	0.457442	0.039*	
C14	0.67580 (19)	0.4579 (4)	0.42552 (8)	0.0366 (5)	
H14	0.716474	0.588155	0.434344	0.044*	
C15	0.39177 (17)	0.2360 (4)	0.44248 (7)	0.0293 (5)	
C16	0.35361 (19)	0.0448 (4)	0.46614 (8)	0.0362 (5)	
H16	0.400371	−0.079872	0.471962	0.043*	
C17	0.2475 (2)	0.0349 (5)	0.48133 (9)	0.0437 (6)	
H17	0.222173	−0.096227	0.497663	0.052*	
C18	0.1787 (2)	0.2137 (5)	0.47289 (9)	0.0471 (7)	
H18	0.106092	0.206011	0.483266	0.057*	
C19	0.2158 (2)	0.4046 (5)	0.44923 (9)	0.0448 (6)	
H19	0.168487	0.528178	0.443247	0.054*	
C20	0.32183 (19)	0.4161 (4)	0.43423 (8)	0.0360 (5)	
H20	0.346918	0.548087	0.418186	0.043*	
C21	0.83900 (19)	0.3060 (5)	0.38057 (10)	0.0484 (7)	
H21	0.861763	0.153443	0.368734	0.058*	
C22	0.9186 (2)	0.3812 (8)	0.41767 (12)	0.0823 (13)	
H22A	0.989972	0.389687	0.402947	0.123*	
H22B	0.898205	0.530877	0.42989	0.123*	
H22C	0.920409	0.273085	0.44482	0.123*	
C23	0.83496 (16)	0.4652 (4)	0.33647 (8)	0.0329 (5)	
N1	0.85361 (16)	0.9339 (4)	0.26431 (8)	0.0319 (4)	
O1	0.83181 (13)	0.3767 (3)	0.29437 (6)	0.0388 (4)	
O2	0.83086 (15)	0.6720 (3)	0.34405 (6)	0.0470 (5)	
F1	0.49883 (12)	−0.0979 (2)	0.38696 (6)	0.0499 (4)	
H1A	0.797 (2)	0.927 (5)	0.2430 (11)	0.045 (7)*	
H1C	0.857 (2)	1.077 (5)	0.2754 (10)	0.044 (7)*	
H1B	0.848 (2)	0.838 (5)	0.2918 (11)	0.046 (8)*	

### (RS)-1-Phenylethan-1-aminium 2-(3-fluoro-4-phenylphenyl)propanoate (II) Atomic displacement parameters (Å^2^)

**Table:**

	*U*^11^	*U*^22^	*U*^33^	*U*^12^	*U*^13^	*U*^23^
C1	0.0316 (11)	0.0356 (13)	0.0316 (11)	0.0004 (10)	0.0037 (9)	0.0034 (10)
C2	0.0384 (13)	0.0474 (15)	0.0391 (13)	0.0060 (11)	−0.0021 (10)	−0.0042 (11)
C3	0.0493 (16)	0.069 (2)	0.0401 (14)	−0.0014 (14)	−0.0093 (11)	−0.0036 (13)
C4	0.0421 (15)	0.074 (2)	0.0642 (18)	0.0028 (15)	−0.0166 (13)	0.0232 (17)
C5	0.0456 (17)	0.0524 (19)	0.104 (3)	0.0130 (14)	−0.0061 (16)	0.0169 (18)
C6	0.0447 (15)	0.0367 (14)	0.0746 (19)	0.0029 (12)	−0.0002 (13)	−0.0020 (13)
C7	0.0342 (12)	0.0346 (12)	0.0315 (11)	−0.0024 (10)	0.0025 (9)	−0.0048 (9)
C8	0.0420 (14)	0.0638 (18)	0.0339 (12)	−0.0147 (13)	−0.0048 (10)	0.0077 (12)
C9	0.0315 (11)	0.0412 (13)	0.0346 (12)	0.0054 (10)	−0.0007 (9)	0.0152 (10)
C10	0.0396 (13)	0.0340 (13)	0.0366 (12)	0.0095 (10)	0.0060 (10)	0.0060 (10)
C11	0.0386 (12)	0.0277 (12)	0.0325 (11)	−0.0003 (9)	0.0001 (9)	0.0020 (9)
C12	0.0331 (11)	0.0295 (12)	0.0226 (10)	0.0023 (9)	−0.0021 (8)	0.0039 (9)
C13	0.0406 (12)	0.0304 (12)	0.0269 (11)	0.0006 (10)	−0.0008 (9)	0.0008 (9)
C14	0.0374 (12)	0.0378 (13)	0.0342 (12)	−0.0058 (10)	−0.0070 (9)	0.0076 (10)
C15	0.0335 (11)	0.0318 (12)	0.0226 (10)	−0.0008 (9)	−0.0015 (8)	−0.0035 (9)
C16	0.0396 (13)	0.0369 (13)	0.0323 (11)	−0.0005 (10)	0.0011 (9)	0.0028 (10)
C17	0.0434 (14)	0.0506 (16)	0.0373 (13)	−0.0098 (12)	0.0033 (10)	0.0033 (12)
C18	0.0326 (13)	0.0670 (19)	0.0418 (14)	−0.0058 (12)	0.0021 (10)	−0.0127 (13)
C19	0.0360 (13)	0.0499 (16)	0.0482 (14)	0.0093 (11)	−0.0061 (11)	−0.0097 (12)
C20	0.0373 (12)	0.0341 (13)	0.0364 (12)	0.0018 (10)	−0.0049 (9)	−0.0014 (10)
C21	0.0325 (13)	0.0591 (17)	0.0536 (15)	0.0087 (12)	0.0033 (11)	0.0232 (13)
C22	0.0402 (16)	0.150 (4)	0.0564 (18)	−0.001 (2)	−0.0051 (14)	0.017 (2)
C23	0.0228 (11)	0.0351 (13)	0.0409 (13)	0.0012 (9)	0.0041 (9)	0.0070 (10)
N1	0.0315 (10)	0.0283 (11)	0.0358 (11)	−0.0018 (8)	0.0007 (8)	−0.0004 (9)
O1	0.0368 (9)	0.0327 (9)	0.0467 (10)	0.0030 (7)	−0.0031 (7)	−0.0041 (7)
O2	0.0644 (12)	0.0327 (10)	0.0445 (10)	−0.0038 (8)	0.0175 (8)	−0.0023 (8)
F1	0.0514 (9)	0.0378 (8)	0.0609 (9)	−0.0053 (7)	0.0103 (7)	−0.0134 (7)

### (RS)-1-Phenylethan-1-aminium 2-(3-fluoro-4-phenylphenyl)propanoate (II) Geometric parameters (Å, º)

**Table:**

C1—C2	1.383 (3)	C12—C15	1.487 (3)
C1—C6	1.386 (4)	C13—C14	1.387 (3)
C1—C7	1.516 (3)	C13—H13	0.95
C2—C3	1.389 (4)	C14—H14	0.95
C2—H2	0.95	C15—C16	1.389 (3)
C3—C4	1.375 (4)	C15—C20	1.390 (3)
C3—H3	0.95	C16—C17	1.388 (3)
C4—C5	1.374 (5)	C16—H16	0.95
C4—H4	0.95	C17—C18	1.376 (4)
C5—C6	1.384 (4)	C17—H17	0.95
C5—H5	0.95	C18—C19	1.383 (4)
C6—H6	0.95	C18—H18	0.95
C7—N1	1.490 (3)	C19—C20	1.386 (3)
C7—C8	1.510 (3)	C19—H19	0.95
C7—H7	1	C20—H20	0.95
C8—H8A	0.98	C21—C22	1.475 (4)
C8—H8B	0.98	C21—C23	1.531 (3)
C8—H8C	0.98	C21—H21	1
C9—C10	1.384 (3)	C22—H22A	0.98
C9—C14	1.394 (4)	C22—H22B	0.98
C9—C21	1.523 (3)	C22—H22C	0.98
C10—C11	1.378 (3)	C23—O2	1.241 (3)
C10—H10	0.95	C23—O1	1.265 (3)
C11—F1	1.358 (3)	N1—H1A	0.91 (3)
C11—C12	1.383 (3)	N1—H1C	0.90 (3)
C12—C13	1.396 (3)	N1—H1B	0.95 (3)
			
C2—C1—C6	118.8 (2)	C12—C13—H13	119.1
C2—C1—C7	121.5 (2)	C13—C14—C9	120.7 (2)
C6—C1—C7	119.7 (2)	C13—C14—H14	119.7
C1—C2—C3	120.6 (2)	C9—C14—H14	119.7
C1—C2—H2	119.7	C16—C15—C20	118.8 (2)
C3—C2—H2	119.7	C16—C15—C12	121.1 (2)
C4—C3—C2	120.0 (3)	C20—C15—C12	120.0 (2)
C4—C3—H3	120	C17—C16—C15	120.3 (2)
C2—C3—H3	120	C17—C16—H16	119.8
C5—C4—C3	119.9 (3)	C15—C16—H16	119.8
C5—C4—H4	120	C18—C17—C16	120.4 (2)
C3—C4—H4	120	C18—C17—H17	119.8
C4—C5—C6	120.2 (3)	C16—C17—H17	119.8
C4—C5—H5	119.9	C17—C18—C19	119.7 (2)
C6—C5—H5	119.9	C17—C18—H18	120.2
C5—C6—C1	120.5 (3)	C19—C18—H18	120.2
C5—C6—H6	119.7	C18—C19—C20	120.1 (2)
C1—C6—H6	119.7	C18—C19—H19	119.9
N1—C7—C8	109.5 (2)	C20—C19—H19	119.9
N1—C7—C1	110.34 (18)	C19—C20—C15	120.5 (2)
C8—C7—C1	112.63 (19)	C19—C20—H20	119.7
N1—C7—H7	108.1	C15—C20—H20	119.7
C8—C7—H7	108.1	C22—C21—C9	114.8 (2)
C1—C7—H7	108.1	C22—C21—C23	111.8 (3)
C7—C8—H8A	109.5	C9—C21—C23	106.63 (18)
C7—C8—H8B	109.5	C22—C21—H21	107.8
H8A—C8—H8B	109.5	C9—C21—H21	107.8
C7—C8—H8C	109.5	C23—C21—H21	107.8
H8A—C8—H8C	109.5	C21—C22—H22A	109.5
H8B—C8—H8C	109.5	C21—C22—H22B	109.5
C10—C9—C14	118.4 (2)	H22A—C22—H22B	109.5
C10—C9—C21	119.8 (2)	C21—C22—H22C	109.5
C14—C9—C21	121.7 (2)	H22A—C22—H22C	109.5
C11—C10—C9	119.5 (2)	H22B—C22—H22C	109.5
C11—C10—H10	120.3	O2—C23—O1	124.0 (2)
C9—C10—H10	120.3	O2—C23—C21	118.3 (2)
F1—C11—C10	117.7 (2)	O1—C23—C21	117.6 (2)
F1—C11—C12	118.2 (2)	C7—N1—H1A	107.0 (17)
C10—C11—C12	124.0 (2)	C7—N1—H1C	110.5 (18)
C11—C12—C13	115.6 (2)	H1A—N1—H1C	107 (2)
C11—C12—C15	122.9 (2)	C7—N1—H1B	110.3 (17)
C13—C12—C15	121.50 (19)	H1A—N1—H1B	115 (2)
C14—C13—C12	121.8 (2)	H1C—N1—H1B	108 (2)
C14—C13—H13	119.1		
			
C6—C1—C2—C3	−0.6 (4)	C10—C9—C14—C13	−0.5 (3)
C7—C1—C2—C3	−177.7 (2)	C21—C9—C14—C13	176.3 (2)
C1—C2—C3—C4	0.8 (4)	C11—C12—C15—C16	50.7 (3)
C2—C3—C4—C5	−0.2 (5)	C13—C12—C15—C16	−129.4 (2)
C3—C4—C5—C6	−0.5 (5)	C11—C12—C15—C20	−131.3 (2)
C4—C5—C6—C1	0.7 (5)	C13—C12—C15—C20	48.6 (3)
C2—C1—C6—C5	−0.1 (4)	C20—C15—C16—C17	−0.2 (3)
C7—C1—C6—C5	177.1 (3)	C12—C15—C16—C17	177.9 (2)
C2—C1—C7—N1	−53.5 (3)	C15—C16—C17—C18	0.4 (4)
C6—C1—C7—N1	129.5 (2)	C16—C17—C18—C19	−0.2 (4)
C2—C1—C7—C8	69.3 (3)	C17—C18—C19—C20	−0.2 (4)
C6—C1—C7—C8	−107.8 (3)	C18—C19—C20—C15	0.4 (4)
C14—C9—C10—C11	0.7 (3)	C16—C15—C20—C19	−0.2 (3)
C21—C9—C10—C11	−176.2 (2)	C12—C15—C20—C19	−178.3 (2)
C9—C10—C11—F1	177.5 (2)	C10—C9—C21—C22	−134.9 (3)
C9—C10—C11—C12	−0.7 (3)	C14—C9—C21—C22	48.3 (4)
F1—C11—C12—C13	−177.81 (18)	C10—C9—C21—C23	100.7 (3)
C10—C11—C12—C13	0.4 (3)	C14—C9—C21—C23	−76.1 (3)
F1—C11—C12—C15	2.1 (3)	C22—C21—C23—O2	−46.8 (3)
C10—C11—C12—C15	−179.7 (2)	C9—C21—C23—O2	79.5 (3)
C11—C12—C13—C14	−0.2 (3)	C22—C21—C23—O1	135.9 (3)
C15—C12—C13—C14	179.93 (19)	C9—C21—C23—O1	−97.8 (3)
C12—C13—C14—C9	0.3 (3)		

### (RS)-1-Phenylethan-1-aminium 2-(3-fluoro-4-phenylphenyl)propanoate (II) Hydrogen-bond geometry (Å, º)

**Table:**

*D*—H···*A*	*D*—H	H···*A*	*D*···*A*	*D*—H···*A*
N1—H1*A*···O1^i^	0.91 (3)	1.91 (3)	2.809 (3)	170 (3)
N1—H1*C*···O1^ii^	0.90 (3)	1.87 (3)	2.758 (3)	168 (3)
N1—H1*B*···O2	0.95 (3)	1.75 (3)	2.693 (3)	176 (3)

Symmetry codes: (i) −*x*+3/2, *y*+1/2, −*z*+1/2; (ii) *x*, *y*+1, *z*.

### (RS)-1-Phenylethan-1-aminium 2-chloro-4-nitrobenzoate (III) Crystal data

**Table:**

C_8_H_12_N^+^·C_7_H_3_ClNO_4_^−^	*F*(000) = 1344
*M**_r_* = 322.74	*D*_x_ = 1.4 Mg m^−^^3^
Monoclinic, *C*2/*c*	Mo *K*α radiation, λ = 0.71073 Å
Hall symbol: -C 2yc	Cell parameters from 8180 reflections
*a* = 15.5817 (7) Å	θ = 2.7–28.3°
*b* = 6.3914 (3) Å	µ = 0.27 mm^−^^1^
*c* = 31.3238 (14) Å	*T* = 173 K
β = 100.998 (2)°	Plate, colourless
*V* = 3062.2 (2) Å^3^	0.47 × 0.35 × 0.08 mm
*Z* = 8	

### (RS)-1-Phenylethan-1-aminium 2-chloro-4-nitrobenzoate (III) Data collection

**Table:**

Bruker D8 Venture Photon CCD area detector diffractometer	3218 reflections with *I* > 2σ(*I*)
Graphite monochromator	*R*_int_ = 0.053
ω scans	θ_max_ = 28.0°, θ_min_ = 2.7°
Absorption correction: integration (XPREP; Bruker, 2016)	*h* = −20→20
*T*_min_ = 0.903, *T*_max_ = 0.979	*k* = −8→8
18885 measured reflections	*l* = −41→41
3709 independent reflections	

### (RS)-1-Phenylethan-1-aminium 2-chloro-4-nitrobenzoate (III) Refinement

**Table:**

Refinement on *F*^2^	0 constraints
Least-squares matrix: full	Hydrogen site location: mixed
*R*[*F*^2^ > 2σ(*F*^2^)] = 0.035	H atoms treated by a mixture of independent and constrained refinement
*wR*(*F*^2^) = 0.091	*w* = 1/[σ^2^(*F*_o_^2^) + (0.046*P*)^2^ + 1.4672*P*] where *P* = (*F*_o_^2^ + 2*F*_c_^2^)/3
*S* = 1.04	(Δ/σ)_max_ = 0.001
3709 reflections	Δρ_max_ = 0.28 e Å^−^^3^
212 parameters	Δρ_min_ = −0.31 e Å^−^^3^
0 restraints	

### (RS)-1-Phenylethan-1-aminium 2-chloro-4-nitrobenzoate (III) Special details

**Table:**

Experimental. Numerical integration absorption corrections based on indexed crystal faces were applied using the XPREP routine (Bruker, 2016)
Geometry. All esds (except the esd in the dihedral angle between two l.s. planes) are estimated using the full covariance matrix. The cell esds are taken into account individually in the estimation of esds in distances, angles and torsion angles; correlations between esds in cell parameters are only used when they are defined by crystal symmetry. An approximate (isotropic) treatment of cell esds is used for estimating esds involving l.s. planes.

### (RS)-1-Phenylethan-1-aminium 2-chloro-4-nitrobenzoate (III) Fractional atomic coordinates and isotropic or equivalent isotropic displacement parameters (Å^2^)

**Table:**

	*x*	*y*	*z*	*U*_iso_*/*U*_eq_	
C1	0.09581 (10)	0.3921 (3)	0.05256 (5)	0.0435 (4)	
H1	0.088661	0.371465	0.022052	0.052*	
C2	0.09009 (10)	0.2257 (3)	0.07959 (5)	0.0440 (4)	
H2	0.079856	0.089053	0.067772	0.053*	
C3	0.09920 (9)	0.2562 (2)	0.12412 (5)	0.0360 (3)	
H3	0.094958	0.140054	0.142536	0.043*	
C4	0.11437 (8)	0.4538 (2)	0.14197 (4)	0.0263 (3)	
C5	0.12131 (11)	0.6209 (2)	0.11467 (5)	0.0412 (3)	
H5	0.132454	0.757299	0.126471	0.049*	
C6	0.11206 (12)	0.5899 (3)	0.07006 (5)	0.0492 (4)	
H6	0.116926	0.705265	0.051546	0.059*	
C7	0.12507 (8)	0.4829 (2)	0.19075 (4)	0.0261 (3)	
H7	0.108406	0.348778	0.20347	0.031*	
C8	0.06945 (9)	0.6569 (2)	0.20441 (4)	0.0353 (3)	
H8A	0.007558	0.627874	0.193002	0.053*	
H8B	0.085549	0.790563	0.192793	0.053*	
H8C	0.079383	0.664261	0.236219	0.053*	
N1	0.21931 (6)	0.52603 (17)	0.20964 (3)	0.0233 (2)	
H1A	0.2343 (10)	0.661 (3)	0.2022 (5)	0.035 (4)*	
H1B	0.2564 (10)	0.429 (3)	0.1998 (5)	0.034 (4)*	
H1C	0.2276 (10)	0.518 (2)	0.2403 (5)	0.033 (4)*	
C9	0.33113 (8)	0.90589 (19)	0.14294 (4)	0.0244 (2)	
C10	0.37797 (7)	0.72139 (19)	0.15321 (4)	0.0236 (2)	
C11	0.39367 (8)	0.5834 (2)	0.12153 (4)	0.0277 (3)	
H11	0.423888	0.455476	0.128894	0.033*	
C12	0.36346 (9)	0.6399 (2)	0.07865 (4)	0.0330 (3)	
C13	0.32140 (11)	0.8263 (2)	0.06657 (4)	0.0386 (3)	
H13	0.303972	0.862985	0.03679	0.046*	
C14	0.30526 (9)	0.9589 (2)	0.09922 (4)	0.0332 (3)	
H14	0.276081	1.087962	0.091646	0.04*	
C15	0.30282 (7)	1.04387 (19)	0.17704 (4)	0.0238 (2)	
N2	0.37830 (10)	0.4929 (2)	0.04459 (4)	0.0453 (3)	
O1	0.25740 (6)	0.95511 (14)	0.20132 (3)	0.0280 (2)	
O2	0.32294 (6)	1.23110 (14)	0.17769 (3)	0.0323 (2)	
O3	0.41721 (10)	0.33069 (19)	0.05555 (4)	0.0595 (4)	
O4	0.35101 (12)	0.5415 (2)	0.00671 (4)	0.0796 (5)	
Cl1	0.42338 (2)	0.66402 (5)	0.20708 (2)	0.02886 (10)	

### (RS)-1-Phenylethan-1-aminium 2-chloro-4-nitrobenzoate (III) Atomic displacement parameters (Å^2^)

**Table:**

	*U*^11^	*U*^22^	*U*^33^	*U*^12^	*U*^13^	*U*^23^
C1	0.0479 (8)	0.0530 (10)	0.0283 (6)	0.0043 (7)	0.0041 (6)	−0.0096 (6)
C2	0.0523 (9)	0.0377 (9)	0.0410 (8)	−0.0035 (7)	0.0058 (7)	−0.0159 (7)
C3	0.0414 (7)	0.0285 (7)	0.0380 (7)	−0.0027 (6)	0.0071 (6)	−0.0027 (6)
C4	0.0260 (5)	0.0264 (7)	0.0264 (6)	−0.0023 (5)	0.0051 (4)	−0.0016 (5)
C5	0.0664 (10)	0.0279 (7)	0.0295 (7)	−0.0040 (7)	0.0095 (6)	−0.0011 (5)
C6	0.0767 (11)	0.0428 (9)	0.0283 (7)	0.0003 (8)	0.0106 (7)	0.0048 (6)
C7	0.0276 (5)	0.0258 (6)	0.0260 (6)	−0.0049 (5)	0.0078 (4)	0.0010 (5)
C8	0.0308 (6)	0.0439 (9)	0.0334 (7)	0.0035 (6)	0.0113 (5)	−0.0021 (6)
N1	0.0283 (5)	0.0208 (5)	0.0221 (5)	−0.0008 (4)	0.0080 (4)	−0.0002 (4)
C9	0.0294 (6)	0.0209 (6)	0.0244 (5)	−0.0018 (4)	0.0093 (4)	−0.0007 (4)
C10	0.0267 (5)	0.0246 (6)	0.0200 (5)	−0.0031 (4)	0.0059 (4)	0.0000 (4)
C11	0.0331 (6)	0.0240 (6)	0.0275 (6)	0.0020 (5)	0.0097 (5)	−0.0007 (5)
C12	0.0474 (7)	0.0300 (7)	0.0247 (6)	0.0032 (6)	0.0145 (5)	−0.0030 (5)
C13	0.0591 (9)	0.0362 (8)	0.0222 (6)	0.0069 (6)	0.0119 (6)	0.0040 (5)
C14	0.0482 (7)	0.0268 (7)	0.0267 (6)	0.0073 (6)	0.0121 (5)	0.0052 (5)
C15	0.0272 (5)	0.0223 (6)	0.0220 (5)	0.0013 (4)	0.0052 (4)	−0.0004 (4)
N2	0.0727 (9)	0.0388 (8)	0.0279 (6)	0.0080 (6)	0.0186 (6)	−0.0049 (5)
O1	0.0368 (5)	0.0249 (5)	0.0251 (4)	−0.0034 (4)	0.0127 (3)	−0.0024 (3)
O2	0.0407 (5)	0.0201 (5)	0.0396 (5)	−0.0024 (4)	0.0167 (4)	−0.0030 (4)
O3	0.0975 (10)	0.0428 (7)	0.0406 (6)	0.0242 (7)	0.0192 (6)	−0.0079 (5)
O4	0.1472 (14)	0.0678 (9)	0.0243 (5)	0.0380 (9)	0.0182 (7)	−0.0036 (6)
Cl1	0.02955 (16)	0.03213 (18)	0.02288 (15)	0.00106 (11)	−0.00007 (11)	−0.00070 (11)

### (RS)-1-Phenylethan-1-aminium 2-chloro-4-nitrobenzoate (III) Geometric parameters (Å, º)

**Table:**

C1—C2	1.373 (2)	N1—H1B	0.940 (16)
C1—C6	1.382 (2)	N1—H1C	0.946 (16)
C1—H1	0.95	C9—C10	1.3919 (17)
C2—C3	1.389 (2)	C9—C14	1.3933 (17)
C2—H2	0.95	C9—C15	1.5143 (16)
C3—C4	1.3833 (19)	C10—C11	1.3837 (17)
C3—H3	0.95	C10—Cl1	1.7397 (11)
C4—C5	1.3848 (19)	C11—C12	1.3836 (18)
C4—C7	1.5167 (16)	C11—H11	0.95
C5—C6	1.391 (2)	C12—C13	1.377 (2)
C5—H5	0.95	C12—N2	1.4726 (17)
C6—H6	0.95	C13—C14	1.3876 (19)
C7—N1	1.5003 (15)	C13—H13	0.95
C7—C8	1.5206 (19)	C14—H14	0.95
C7—H7	1	C15—O2	1.2362 (15)
C8—H8A	0.98	C15—O1	1.2675 (14)
C8—H8B	0.98	N2—O3	1.2171 (17)
C8—H8C	0.98	N2—O4	1.2216 (17)
N1—H1A	0.937 (17)		
			
C2—C1—C6	119.52 (13)	C7—N1—H1A	110.0 (9)
C2—C1—H1	120.2	C7—N1—H1B	111.4 (9)
C6—C1—H1	120.2	H1A—N1—H1B	109.4 (13)
C1—C2—C3	120.29 (14)	C7—N1—H1C	108.9 (9)
C1—C2—H2	119.9	H1A—N1—H1C	107.9 (13)
C3—C2—H2	119.9	H1B—N1—H1C	109.0 (13)
C4—C3—C2	120.72 (14)	C10—C9—C14	118.28 (11)
C4—C3—H3	119.6	C10—C9—C15	122.74 (10)
C2—C3—H3	119.6	C14—C9—C15	118.89 (11)
C3—C4—C5	118.82 (12)	C11—C10—C9	122.05 (11)
C3—C4—C7	119.75 (12)	C11—C10—Cl1	117.81 (9)
C5—C4—C7	121.41 (12)	C9—C10—Cl1	120.07 (9)
C4—C5—C6	120.33 (14)	C12—C11—C10	117.13 (12)
C4—C5—H5	119.8	C12—C11—H11	121.4
C6—C5—H5	119.8	C10—C11—H11	121.4
C1—C6—C5	120.30 (15)	C13—C12—C11	123.24 (12)
C1—C6—H6	119.8	C13—C12—N2	119.02 (12)
C5—C6—H6	119.8	C11—C12—N2	117.74 (12)
N1—C7—C4	109.33 (9)	C12—C13—C14	118.00 (12)
N1—C7—C8	108.79 (10)	C12—C13—H13	121
C4—C7—C8	114.45 (11)	C14—C13—H13	121
N1—C7—H7	108	C13—C14—C9	121.11 (12)
C4—C7—H7	108	C13—C14—H14	119.4
C8—C7—H7	108	C9—C14—H14	119.4
C7—C8—H8A	109.5	O2—C15—O1	126.32 (11)
C7—C8—H8B	109.5	O2—C15—C9	117.92 (10)
H8A—C8—H8B	109.5	O1—C15—C9	115.71 (11)
C7—C8—H8C	109.5	O3—N2—O4	123.54 (13)
H8A—C8—H8C	109.5	O3—N2—C12	118.55 (12)
H8B—C8—H8C	109.5	O4—N2—C12	117.91 (13)
			
C6—C1—C2—C3	1.0 (2)	Cl1—C10—C11—C12	−174.67 (10)
C1—C2—C3—C4	−0.2 (2)	C10—C11—C12—C13	1.7 (2)
C2—C3—C4—C5	−0.7 (2)	C10—C11—C12—N2	−178.93 (12)
C2—C3—C4—C7	−179.33 (13)	C11—C12—C13—C14	−3.0 (2)
C3—C4—C5—C6	0.8 (2)	N2—C12—C13—C14	177.66 (14)
C7—C4—C5—C6	179.39 (14)	C12—C13—C14—C9	0.3 (2)
C2—C1—C6—C5	−0.9 (3)	C10—C9—C14—C13	3.5 (2)
C4—C5—C6—C1	0.0 (3)	C15—C9—C14—C13	−173.03 (13)
C3—C4—C7—N1	107.18 (13)	C10—C9—C15—O2	126.58 (13)
C5—C4—C7—N1	−71.41 (16)	C14—C9—C15—O2	−57.09 (16)
C3—C4—C7—C8	−130.51 (13)	C10—C9—C15—O1	−55.96 (15)
C5—C4—C7—C8	50.90 (17)	C14—C9—C15—O1	120.37 (13)
C14—C9—C10—C11	−4.84 (18)	C13—C12—N2—O3	178.42 (16)
C15—C9—C10—C11	171.52 (11)	C11—C12—N2—O3	−0.9 (2)
C14—C9—C10—Cl1	172.07 (10)	C13—C12—N2—O4	−1.3 (2)
C15—C9—C10—Cl1	−11.58 (16)	C11—C12—N2—O4	179.34 (16)
C9—C10—C11—C12	2.30 (18)		

### (RS)-1-Phenylethan-1-aminium 2-chloro-4-nitrobenzoate (III) Hydrogen-bond geometry (Å, º)

**Table:**

*D*—H···*A*	*D*—H	H···*A*	*D*···*A*	*D*—H···*A*
N1—H1*A*···O1	0.94 (2)	1.91 (2)	2.829 (1)	165 (1)
N1—H1*B*···O2^i^	0.94 (2)	1.85 (2)	2.789 (1)	176 (1)
N1—H1*C*···O1^ii^	0.95 (2)	1.84 (2)	2.780 (1)	170 (1)

Symmetry codes: (i) *x*, *y*−1, *z*; (ii) −*x*+1/2, *y*−1/2, −*z*+1/2.

### (RS)-1-Phenylethan-1-aminium 4-iodobenzoate (IV) Crystal data

**Table:**

C_8_H_12_N^+^·C_7_H_4_IO_2_^−^	*F*(000) = 728
*M**_r_* = 369.19	*D*_x_ = 1.697 Mg m^−^^3^
Monoclinic, *P*2_1_/*n*	Mo *K*α radiation, λ = 0.71073 Å
Hall symbol: -P 2yn	Cell parameters from 9122 reflections
*a* = 9.7224 (5) Å	θ = 3.3–28.3°
*b* = 6.0571 (3) Å	µ = 2.21 mm^−^^1^
*c* = 24.8767 (12) Å	*T* = 173 K
β = 99.527 (2)°	Plate, orange
*V* = 1444.77 (12) Å^3^	0.68 × 0.16 × 0.04 mm
*Z* = 4	

### (RS)-1-Phenylethan-1-aminium 4-iodobenzoate (IV) Data collection

**Table:**

Bruker D8 Venture Photon CCD area detector diffractometer	3109 reflections with *I* > 2σ(*I*)
Graphite monochromator	*R*_int_ = 0.051
ω scans	θ_max_ = 28.0°, θ_min_ = 3.0°
Absorption correction: integration (XPREP; Bruker, 2016)	*h* = −12→12
*T*_min_ = 0.508, *T*_max_ = 0.928	*k* = −8→8
30978 measured reflections	*l* = −32→32
3463 independent reflections	

### (RS)-1-Phenylethan-1-aminium 4-iodobenzoate (IV) Refinement

**Table:**

Refinement on *F*^2^	0 constraints
Least-squares matrix: full	Hydrogen site location: mixed
*R*[*F*^2^ > 2σ(*F*^2^)] = 0.026	H atoms treated by a mixture of independent and constrained refinement
*wR*(*F*^2^) = 0.057	*w* = 1/[σ^2^(*F*_o_^2^) + (0.0189*P*)^2^ + 1.5669*P*] where *P* = (*F*_o_^2^ + 2*F*_c_^2^)/3
*S* = 1.13	(Δ/σ)_max_ = 0.001
3463 reflections	Δρ_max_ = 0.74 e Å^−^^3^
185 parameters	Δρ_min_ = −0.38 e Å^−^^3^
0 restraints	

### (RS)-1-Phenylethan-1-aminium 4-iodobenzoate (IV) Special details

**Table:**

Experimental. Numerical integration absorption corrections based on indexed crystal faces were applied using the XPREP routine (Bruker, 2016)
Geometry. All esds (except the esd in the dihedral angle between two l.s. planes) are estimated using the full covariance matrix. The cell esds are taken into account individually in the estimation of esds in distances, angles and torsion angles; correlations between esds in cell parameters are only used when they are defined by crystal symmetry. An approximate (isotropic) treatment of cell esds is used for estimating esds involving l.s. planes.

### (RS)-1-Phenylethan-1-aminium 4-iodobenzoate (IV) Fractional atomic coordinates and isotropic or equivalent isotropic displacement parameters (Å^2^)

**Table:**

	*x*	*y*	*z*	*U*_iso_*/*U*_eq_	
C1	0.4776 (2)	0.5625 (4)	0.30540 (9)	0.0228 (4)	
C2	0.5527 (2)	0.3668 (4)	0.30591 (9)	0.0264 (5)	
H2	0.600702	0.334421	0.276565	0.032*	
C3	0.5584 (2)	0.2182 (4)	0.34868 (10)	0.0273 (5)	
H3	0.611349	0.086327	0.348888	0.033*	
C4	0.4864 (2)	0.2631 (4)	0.39109 (10)	0.0277 (5)	
H4	0.488952	0.161314	0.420293	0.033*	
C5	0.4111 (2)	0.4563 (4)	0.39071 (9)	0.0292 (5)	
H5	0.361284	0.486833	0.419662	0.035*	
C6	0.4075 (2)	0.6059 (4)	0.34849 (9)	0.0273 (5)	
H6	0.356522	0.739462	0.348997	0.033*	
C7	0.4644 (2)	0.7258 (4)	0.25903 (10)	0.0263 (5)	
H7	0.440241	0.872568	0.273366	0.032*	
C8	0.3496 (3)	0.6640 (5)	0.21243 (11)	0.0361 (6)	
H8A	0.366585	0.515084	0.199602	0.054*	
H8B	0.348559	0.769447	0.182486	0.054*	
H8C	0.259354	0.667562	0.225099	0.054*	
N1	0.5992 (2)	0.7512 (3)	0.23771 (8)	0.0214 (4)	
H1A	0.621 (3)	0.633 (5)	0.2208 (10)	0.023 (6)*	
H1B	0.591 (3)	0.858 (5)	0.2159 (11)	0.026 (7)*	
H1C	0.670 (3)	0.780 (4)	0.2662 (12)	0.031 (7)*	
C9	0.5029 (2)	0.3948 (4)	0.10780 (8)	0.0204 (4)	
C10	0.5399 (2)	0.6044 (4)	0.09238 (9)	0.0233 (4)	
H10	0.623639	0.670573	0.110297	0.028*	
C11	0.4551 (2)	0.7177 (4)	0.05091 (9)	0.0249 (4)	
H11	0.480966	0.860353	0.040232	0.03*	
C12	0.3327 (2)	0.6210 (4)	0.02534 (8)	0.0240 (4)	
C13	0.2946 (2)	0.4109 (4)	0.03976 (9)	0.0279 (5)	
H13	0.210789	0.344932	0.021782	0.034*	
C14	0.3806 (2)	0.2995 (4)	0.08068 (9)	0.0246 (4)	
H14	0.355719	0.155055	0.0905	0.029*	
C15	0.5894 (2)	0.2737 (4)	0.15462 (9)	0.0220 (4)	
O1	0.68527 (16)	0.3835 (3)	0.18395 (6)	0.0249 (3)	
O2	0.5591 (2)	0.0792 (3)	0.16235 (7)	0.0375 (4)	
I1	0.19967 (2)	0.79494 (3)	−0.03513 (2)	0.03359 (6)	

### (RS)-1-Phenylethan-1-aminium 4-iodobenzoate (IV) Atomic displacement parameters (Å^2^)

**Table:**

	*U*^11^	*U*^22^	*U*^33^	*U*^12^	*U*^13^	*U*^23^
C1	0.0175 (10)	0.0242 (11)	0.0258 (11)	−0.0025 (8)	0.0005 (8)	0.0018 (9)
C2	0.0263 (11)	0.0296 (12)	0.0245 (11)	0.0037 (9)	0.0076 (9)	0.0029 (9)
C3	0.0279 (11)	0.0245 (11)	0.0292 (12)	0.0029 (9)	0.0039 (9)	0.0025 (9)
C4	0.0262 (11)	0.0326 (13)	0.0239 (11)	−0.0014 (9)	0.0029 (9)	0.0042 (9)
C5	0.0254 (11)	0.0395 (14)	0.0236 (11)	0.0012 (10)	0.0063 (9)	−0.0019 (10)
C6	0.0226 (11)	0.0278 (12)	0.0308 (12)	0.0048 (9)	0.0023 (9)	−0.0002 (10)
C7	0.0219 (10)	0.0269 (11)	0.0300 (12)	0.0042 (9)	0.0036 (9)	0.0039 (9)
C8	0.0250 (12)	0.0472 (16)	0.0339 (13)	0.0017 (11)	−0.0019 (10)	0.0057 (12)
N1	0.0213 (9)	0.0191 (10)	0.0224 (9)	−0.0012 (7)	−0.0009 (7)	0.0037 (8)
C9	0.0229 (10)	0.0205 (10)	0.0183 (9)	0.0012 (8)	0.0046 (8)	0.0004 (8)
C10	0.0251 (11)	0.0214 (11)	0.0224 (10)	−0.0032 (9)	0.0010 (8)	0.0002 (9)
C11	0.0330 (12)	0.0201 (10)	0.0219 (10)	0.0008 (9)	0.0056 (9)	0.0036 (9)
C12	0.0290 (11)	0.0242 (11)	0.0178 (10)	0.0077 (9)	0.0010 (8)	−0.0010 (9)
C13	0.0263 (11)	0.0264 (12)	0.0289 (12)	−0.0016 (9)	−0.0017 (9)	−0.0023 (10)
C14	0.0264 (11)	0.0193 (10)	0.0275 (11)	−0.0030 (9)	0.0031 (9)	0.0017 (9)
C15	0.0258 (10)	0.0216 (10)	0.0189 (10)	0.0019 (8)	0.0047 (8)	0.0020 (8)
O1	0.0251 (8)	0.0259 (8)	0.0218 (7)	0.0011 (6)	−0.0020 (6)	0.0004 (6)
O2	0.0508 (11)	0.0234 (9)	0.0334 (9)	−0.0059 (8)	−0.0069 (8)	0.0109 (7)
I1	0.03935 (10)	0.03639 (10)	0.02241 (9)	0.01375 (7)	−0.00260 (6)	0.00224 (6)

### (RS)-1-Phenylethan-1-aminium 4-iodobenzoate (IV) Geometric parameters (Å, º)

**Table:**

C1—C6	1.387 (3)	N1—H1A	0.88 (3)
C1—C2	1.391 (3)	N1—H1B	0.84 (3)
C1—C7	1.509 (3)	N1—H1C	0.92 (3)
C2—C3	1.388 (3)	C9—C10	1.390 (3)
C2—H2	0.95	C9—C14	1.392 (3)
C3—C4	1.386 (3)	C9—C15	1.509 (3)
C3—H3	0.95	C10—C11	1.391 (3)
C4—C5	1.380 (3)	C10—H10	0.95
C4—H4	0.95	C11—C12	1.383 (3)
C5—C6	1.383 (3)	C11—H11	0.95
C5—H5	0.95	C12—C13	1.389 (3)
C6—H6	0.95	C12—I1	2.098 (2)
C7—N1	1.501 (3)	C13—C14	1.382 (3)
C7—C8	1.517 (3)	C13—H13	0.95
C7—H7	1	C14—H14	0.95
C8—H8A	0.98	C15—O2	1.237 (3)
C8—H8B	0.98	C15—O1	1.272 (3)
C8—H8C	0.98		
			
C6—C1—C2	118.6 (2)	H8B—C8—H8C	109.5
C6—C1—C7	118.4 (2)	C7—N1—H1A	112.5 (17)
C2—C1—C7	123.0 (2)	C7—N1—H1B	108.3 (18)
C3—C2—C1	120.9 (2)	H1A—N1—H1B	109 (2)
C3—C2—H2	119.5	C7—N1—H1C	109.4 (17)
C1—C2—H2	119.5	H1A—N1—H1C	108 (2)
C4—C3—C2	119.7 (2)	H1B—N1—H1C	110 (2)
C4—C3—H3	120.2	C10—C9—C14	118.9 (2)
C2—C3—H3	120.2	C10—C9—C15	121.37 (19)
C5—C4—C3	119.7 (2)	C14—C9—C15	119.64 (19)
C5—C4—H4	120.1	C9—C10—C11	120.5 (2)
C3—C4—H4	120.1	C9—C10—H10	119.8
C4—C5—C6	120.5 (2)	C11—C10—H10	119.8
C4—C5—H5	119.8	C12—C11—C10	119.4 (2)
C6—C5—H5	119.8	C12—C11—H11	120.3
C5—C6—C1	120.6 (2)	C10—C11—H11	120.3
C5—C6—H6	119.7	C11—C12—C13	121.0 (2)
C1—C6—H6	119.7	C11—C12—I1	119.89 (17)
N1—C7—C1	111.61 (18)	C13—C12—I1	119.08 (16)
N1—C7—C8	109.3 (2)	C14—C13—C12	118.9 (2)
C1—C7—C8	112.4 (2)	C14—C13—H13	120.6
N1—C7—H7	107.8	C12—C13—H13	120.6
C1—C7—H7	107.8	C13—C14—C9	121.2 (2)
C8—C7—H7	107.8	C13—C14—H14	119.4
C7—C8—H8A	109.5	C9—C14—H14	119.4
C7—C8—H8B	109.5	O2—C15—O1	125.4 (2)
H8A—C8—H8B	109.5	O2—C15—C9	117.8 (2)
C7—C8—H8C	109.5	O1—C15—C9	116.76 (19)
H8A—C8—H8C	109.5		
			
C6—C1—C2—C3	−0.4 (3)	C15—C9—C10—C11	−176.7 (2)
C7—C1—C2—C3	−178.0 (2)	C9—C10—C11—C12	0.6 (3)
C1—C2—C3—C4	1.1 (4)	C10—C11—C12—C13	−1.2 (3)
C2—C3—C4—C5	−0.7 (4)	C10—C11—C12—I1	177.56 (16)
C3—C4—C5—C6	−0.3 (4)	C11—C12—C13—C14	0.6 (3)
C4—C5—C6—C1	1.1 (4)	I1—C12—C13—C14	−178.18 (17)
C2—C1—C6—C5	−0.7 (3)	C12—C13—C14—C9	0.7 (3)
C7—C1—C6—C5	177.0 (2)	C10—C9—C14—C13	−1.3 (3)
C6—C1—C7—N1	142.4 (2)	C15—C9—C14—C13	176.2 (2)
C2—C1—C7—N1	−40.0 (3)	C10—C9—C15—O2	−172.3 (2)
C6—C1—C7—C8	−94.4 (3)	C14—C9—C15—O2	10.3 (3)
C2—C1—C7—C8	83.2 (3)	C10—C9—C15—O1	9.7 (3)
C14—C9—C10—C11	0.7 (3)	C14—C9—C15—O1	−167.7 (2)

### (RS)-1-Phenylethan-1-aminium 4-iodobenzoate (IV) Hydrogen-bond geometry (Å, º)

**Table:**

*D*—H···*A*	*D*—H	H···*A*	*D*···*A*	*D*—H···*A*
N1—H1*A*···O1	0.88 (3)	1.92 (3)	2.796 (3)	175 (2)
N1—H1*B*···O2^i^	0.84 (3)	1.88 (3)	2.715 (3)	174 (3)
N1—H1*C*···O1^ii^	0.92 (3)	1.83 (3)	2.735 (2)	169 (3)

Symmetry codes: (i) *x*, *y*+1, *z*; (ii) −*x*+3/2, *y*+1/2, −*z*+1/2.

### (S)-1-Cyclohexylethan-1-aminium 2-chloro-4-nitrobenzoate (V) Crystal data

**Table:**

C_8_H_18_N^+^·C_7_H_3_ClNO_4_^−^	*F*(000) = 696
*M**_r_* = 328.79	*D*_x_ = 1.383 Mg m^−^^3^
Monoclinic, *C*2	Mo *K*α radiation, λ = 0.71073 Å
Hall symbol: C 2y	Cell parameters from 8605 reflections
*a* = 16.2280 (15) Å	θ = 3.2–31.0°
*b* = 6.4392 (5) Å	µ = 0.26 mm^−^^1^
*c* = 15.5937 (15) Å	*T* = 173 K
β = 104.289 (4)°	Plate, colourless
*V* = 1579.1 (2) Å^3^	0.51 × 0.39 × 0.06 mm
*Z* = 4	

### (S)-1-Cyclohexylethan-1-aminium 2-chloro-4-nitrobenzoate (V) Data collection

**Table:**

Bruker D8 Venture Photon CCD area detector diffractometer	3587 reflections with *I* > 2σ(*I*)
Graphite monochromator	*R*_int_ = 0.045
ω scans	θ_max_ = 28.0°, θ_min_ = 3.2°
Absorption correction: integration (XPREP; Bruker, 2016)	*h* = −21→21
*T*_min_ = 0.910, *T*_max_ = 0.988	*k* = −8→8
15055 measured reflections	*l* = −20→20
3837 independent reflections	

### (S)-1-Cyclohexylethan-1-aminium 2-chloro-4-nitrobenzoate (V) Refinement

**Table:**

Refinement on *F*^2^	Hydrogen site location: mixed
Least-squares matrix: full	H atoms treated by a mixture of independent and constrained refinement
*R*[*F*^2^ > 2σ(*F*^2^)] = 0.032	*w* = 1/[σ^2^(*F*_o_^2^) + (0.0364*P*)^2^ + 0.5174*P*] where *P* = (*F*_o_^2^ + 2*F*_c_^2^)/3
*wR*(*F*^2^) = 0.071	(Δ/σ)_max_ = 0.004
*S* = 1.04	Δρ_max_ = 0.20 e Å^−^^3^
3837 reflections	Δρ_min_ = −0.18 e Å^−^^3^
211 parameters	Absolute structure: Flack *x* determined using 1512 quotients [(*I*^+^)-(*I*^-^)]/[(*I*^+^)+(*I*^-^)] (Parsons *et al.*, 2013).
1 restraint	Absolute structure parameter: −0.031 (19)
0 constraints	

### (S)-1-Cyclohexylethan-1-aminium 2-chloro-4-nitrobenzoate (V) Special details

**Table:**

Experimental. Numerical integration absorption corrections based on indexed crystal faces were applied using the XPREP routine (Bruker, 2016)
Geometry. All esds (except the esd in the dihedral angle between two l.s. planes) are estimated using the full covariance matrix. The cell esds are taken into account individually in the estimation of esds in distances, angles and torsion angles; correlations between esds in cell parameters are only used when they are defined by crystal symmetry. An approximate (isotropic) treatment of cell esds is used for estimating esds involving l.s. planes.

### (S)-1-Cyclohexylethan-1-aminium 2-chloro-4-nitrobenzoate (V) Fractional atomic coordinates and isotropic or equivalent isotropic displacement parameters (Å^2^)

**Table:**

	*x*	*y*	*z*	*U*_iso_*/*U*_eq_	
C1	0.92062 (12)	−0.0254 (3)	0.72741 (12)	0.0222 (4)	
H1	0.983347	−0.000692	0.746046	0.027*	
C2	0.87885 (13)	0.1383 (4)	0.77418 (11)	0.0276 (4)	
H2A	0.816318	0.121419	0.755933	0.033*	
H2B	0.892781	0.278499	0.755797	0.033*	
C3	0.90886 (14)	0.1200 (4)	0.87506 (12)	0.0298 (4)	
H3A	0.97047	0.150661	0.894021	0.036*	
H3B	0.878531	0.223348	0.902958	0.036*	
C4	0.89238 (14)	−0.0959 (4)	0.90568 (13)	0.0306 (5)	
H4A	0.830328	−0.121076	0.892148	0.037*	
H4B	0.915094	−0.106021	0.97058	0.037*	
C5	0.93408 (15)	−0.2602 (4)	0.86040 (14)	0.0324 (5)	
H5A	0.919488	−0.39978	0.878797	0.039*	
H5B	0.996635	−0.244287	0.879238	0.039*	
C6	0.90493 (14)	−0.2421 (3)	0.75965 (13)	0.0278 (4)	
H6A	0.935834	−0.345333	0.73236	0.033*	
H6B	0.843477	−0.274321	0.740347	0.033*	
C7	0.89437 (13)	−0.0078 (3)	0.62578 (12)	0.0245 (4)	
H7	0.913554	−0.13714	0.600976	0.029*	
C8	0.93451 (14)	0.1751 (4)	0.59025 (14)	0.0345 (5)	
H8A	0.996473	0.168179	0.612246	0.052*	
H8B	0.91942	0.170527	0.525424	0.052*	
H8C	0.913611	0.304811	0.61009	0.052*	
C9	0.67604 (12)	0.3841 (3)	0.70870 (12)	0.0195 (4)	
C10	0.63111 (11)	0.1984 (3)	0.68641 (12)	0.0199 (4)	
C11	0.61854 (12)	0.0605 (3)	0.74977 (13)	0.0224 (4)	
H11	0.589347	−0.067073	0.733844	0.027*	
C12	0.65033 (12)	0.1160 (3)	0.83759 (12)	0.0236 (4)	
C13	0.69029 (14)	0.3020 (3)	0.86335 (13)	0.0265 (4)	
H13	0.708769	0.338067	0.924157	0.032*	
C14	0.70281 (13)	0.4352 (3)	0.79816 (12)	0.0244 (4)	
H14	0.730308	0.564495	0.814713	0.029*	
C15	0.70359 (12)	0.5207 (3)	0.64162 (12)	0.0206 (4)	
N1	0.79918 (11)	0.0076 (3)	0.59071 (10)	0.0213 (3)	
N2	0.64113 (13)	−0.0332 (3)	0.90616 (12)	0.0327 (4)	
O1	0.74062 (9)	0.4280 (2)	0.59039 (9)	0.0255 (3)	
O2	0.69186 (10)	0.7100 (2)	0.64544 (10)	0.0288 (3)	
O3	0.61108 (15)	−0.2043 (3)	0.88266 (13)	0.0522 (5)	
O4	0.66539 (14)	0.0207 (3)	0.98356 (11)	0.0488 (5)	
Cl1	0.58672 (3)	0.13764 (8)	0.57637 (3)	0.03040 (13)	
H1A	0.7792 (15)	0.139 (5)	0.5991 (15)	0.030 (6)*	
H1B	0.7824 (16)	−0.011 (4)	0.5295 (17)	0.032 (6)*	
H1C	0.7717 (16)	−0.091 (4)	0.6117 (17)	0.028 (6)*	

### (S)-1-Cyclohexylethan-1-aminium 2-chloro-4-nitrobenzoate (V) Atomic displacement parameters (Å^2^)

**Table:**

	*U*^11^	*U*^22^	*U*^33^	*U*^12^	*U*^13^	*U*^23^
C1	0.0220 (9)	0.0239 (9)	0.0206 (9)	0.0011 (8)	0.0051 (7)	−0.0010 (7)
C2	0.0404 (10)	0.0216 (8)	0.0208 (8)	0.0048 (10)	0.0076 (7)	−0.0005 (9)
C3	0.0385 (10)	0.0301 (11)	0.0204 (8)	0.0028 (10)	0.0063 (8)	−0.0052 (9)
C4	0.0323 (11)	0.0393 (13)	0.0199 (9)	0.0020 (10)	0.0060 (8)	0.0038 (9)
C5	0.0374 (12)	0.0304 (11)	0.0271 (10)	0.0064 (10)	0.0033 (9)	0.0070 (8)
C6	0.0328 (11)	0.0233 (10)	0.0259 (10)	0.0036 (9)	0.0047 (8)	−0.0001 (8)
C7	0.0259 (10)	0.0284 (10)	0.0201 (9)	0.0008 (8)	0.0078 (7)	−0.0020 (8)
C8	0.0345 (11)	0.0439 (15)	0.0270 (10)	−0.0082 (10)	0.0114 (8)	0.0029 (9)
C9	0.0203 (8)	0.0188 (9)	0.0197 (9)	0.0028 (7)	0.0054 (7)	0.0007 (7)
C10	0.0181 (8)	0.0231 (9)	0.0170 (8)	0.0015 (7)	0.0013 (7)	−0.0010 (6)
C11	0.0206 (9)	0.0211 (9)	0.0260 (9)	−0.0004 (7)	0.0068 (7)	0.0002 (7)
C12	0.0239 (8)	0.0269 (10)	0.0227 (8)	0.0034 (9)	0.0106 (7)	0.0062 (8)
C13	0.0304 (10)	0.0330 (11)	0.0168 (9)	0.0004 (9)	0.0075 (8)	−0.0016 (8)
C14	0.0300 (10)	0.0237 (9)	0.0204 (9)	−0.0036 (8)	0.0075 (8)	−0.0047 (7)
C15	0.0210 (9)	0.0216 (10)	0.0178 (8)	−0.0005 (8)	0.0021 (7)	0.0015 (7)
N1	0.0278 (9)	0.0195 (8)	0.0175 (8)	−0.0011 (7)	0.0073 (6)	0.0000 (6)
N2	0.0385 (10)	0.0340 (10)	0.0309 (10)	0.0101 (8)	0.0187 (8)	0.0118 (8)
O1	0.0337 (8)	0.0258 (7)	0.0195 (6)	0.0030 (6)	0.0112 (6)	0.0026 (6)
O2	0.0376 (8)	0.0197 (7)	0.0322 (8)	0.0007 (6)	0.0143 (7)	0.0031 (6)
O3	0.0801 (15)	0.0340 (10)	0.0509 (11)	−0.0100 (10)	0.0321 (10)	0.0099 (8)
O4	0.0711 (13)	0.0519 (11)	0.0261 (8)	0.0079 (10)	0.0171 (8)	0.0137 (8)
Cl1	0.0323 (2)	0.0334 (2)	0.0200 (2)	−0.0058 (2)	−0.00398 (16)	−0.0016 (2)

### (S)-1-Cyclohexylethan-1-aminium 2-chloro-4-nitrobenzoate (V) Geometric parameters (Å, º)

**Table:**

C1—C6	1.525 (3)	C8—H8B	0.98
C1—C2	1.532 (3)	C8—H8C	0.98
C1—C7	1.540 (3)	C9—C14	1.394 (3)
C1—H1	1	C9—C10	1.399 (3)
C2—C3	1.532 (2)	C9—C15	1.516 (3)
C2—H2A	0.99	C10—C11	1.381 (3)
C2—H2B	0.99	C10—Cl1	1.7340 (18)
C3—C4	1.515 (3)	C11—C12	1.386 (3)
C3—H3A	0.99	C11—H11	0.95
C3—H3B	0.99	C12—C13	1.374 (3)
C4—C5	1.520 (3)	C12—N2	1.472 (3)
C4—H4A	0.99	C13—C14	1.383 (3)
C4—H4B	0.99	C13—H13	0.95
C5—C6	1.529 (3)	C14—H14	0.95
C5—H5A	0.99	C15—O2	1.238 (2)
C5—H5B	0.99	C15—O1	1.262 (2)
C6—H6A	0.99	N1—H1A	0.93 (3)
C6—H6B	0.99	N1—H1B	0.93 (3)
C7—N1	1.509 (3)	N1—H1C	0.88 (3)
C7—C8	1.515 (3)	N2—O4	1.224 (3)
C7—H7	1	N2—O3	1.224 (3)
C8—H8A	0.98		
			
C6—C1—C2	110.06 (16)	N1—C7—H7	107.6
C6—C1—C7	112.35 (16)	C8—C7—H7	107.6
C2—C1—C7	113.37 (16)	C1—C7—H7	107.6
C6—C1—H1	106.9	C7—C8—H8A	109.5
C2—C1—H1	106.9	C7—C8—H8B	109.5
C7—C1—H1	106.9	H8A—C8—H8B	109.5
C1—C2—C3	111.71 (17)	C7—C8—H8C	109.5
C1—C2—H2A	109.3	H8A—C8—H8C	109.5
C3—C2—H2A	109.3	H8B—C8—H8C	109.5
C1—C2—H2B	109.3	C14—C9—C10	117.70 (17)
C3—C2—H2B	109.3	C14—C9—C15	118.77 (17)
H2A—C2—H2B	107.9	C10—C9—C15	123.29 (17)
C4—C3—C2	110.99 (18)	C11—C10—C9	122.15 (17)
C4—C3—H3A	109.4	C11—C10—Cl1	117.66 (15)
C2—C3—H3A	109.4	C9—C10—Cl1	120.17 (14)
C4—C3—H3B	109.4	C10—C11—C12	117.15 (18)
C2—C3—H3B	109.4	C10—C11—H11	121.4
H3A—C3—H3B	108	C12—C11—H11	121.4
C3—C4—C5	111.02 (17)	C13—C12—C11	123.21 (18)
C3—C4—H4A	109.4	C13—C12—N2	118.79 (17)
C5—C4—H4A	109.4	C11—C12—N2	118.00 (19)
C3—C4—H4B	109.4	C12—C13—C14	118.03 (17)
C5—C4—H4B	109.4	C12—C13—H13	121
H4A—C4—H4B	108	C14—C13—H13	121
C4—C5—C6	111.33 (17)	C13—C14—C9	121.56 (19)
C4—C5—H5A	109.4	C13—C14—H14	119.2
C6—C5—H5A	109.4	C9—C14—H14	119.2
C4—C5—H5B	109.4	O2—C15—O1	126.8 (2)
C6—C5—H5B	109.4	O2—C15—C9	117.60 (18)
H5A—C5—H5B	108	O1—C15—C9	115.48 (17)
C1—C6—C5	111.95 (17)	C7—N1—H1A	111.8 (15)
C1—C6—H6A	109.2	C7—N1—H1B	112.1 (15)
C5—C6—H6A	109.2	H1A—N1—H1B	104 (2)
C1—C6—H6B	109.2	C7—N1—H1C	112.6 (17)
C5—C6—H6B	109.2	H1A—N1—H1C	112 (2)
H6A—C6—H6B	107.9	H1B—N1—H1C	104 (2)
N1—C7—C8	108.09 (17)	O4—N2—O3	123.90 (19)
N1—C7—C1	112.08 (15)	O4—N2—C12	117.8 (2)
C8—C7—C1	113.51 (17)	O3—N2—C12	118.32 (19)
			
C6—C1—C2—C3	55.1 (2)	Cl1—C10—C11—C12	−176.58 (14)
C7—C1—C2—C3	−178.10 (17)	C10—C11—C12—C13	2.3 (3)
C1—C2—C3—C4	−56.3 (2)	C10—C11—C12—N2	−177.19 (17)
C2—C3—C4—C5	56.1 (2)	C11—C12—C13—C14	−3.2 (3)
C3—C4—C5—C6	−55.7 (2)	N2—C12—C13—C14	176.29 (18)
C2—C1—C6—C5	−54.7 (2)	C12—C13—C14—C9	0.0 (3)
C7—C1—C6—C5	177.98 (16)	C10—C9—C14—C13	3.9 (3)
C4—C5—C6—C1	55.5 (2)	C15—C9—C14—C13	−170.65 (18)
C6—C1—C7—N1	76.3 (2)	C14—C9—C15—O2	−50.9 (3)
C2—C1—C7—N1	−49.2 (2)	C10—C9—C15—O2	134.9 (2)
C6—C1—C7—C8	−160.87 (18)	C14—C9—C15—O1	125.41 (19)
C2—C1—C7—C8	73.6 (2)	C10—C9—C15—O1	−48.8 (3)
C14—C9—C10—C11	−4.8 (3)	C13—C12—N2—O4	4.1 (3)
C15—C9—C10—C11	169.42 (18)	C11—C12—N2—O4	−176.37 (19)
C14—C9—C10—Cl1	173.55 (15)	C13—C12—N2—O3	−174.7 (2)
C15—C9—C10—Cl1	−12.2 (3)	C11—C12—N2—O3	4.8 (3)
C9—C10—C11—C12	1.9 (3)		

### (S)-1-Cyclohexylethan-1-aminium 2-chloro-4-nitrobenzoate (V) Hydrogen-bond geometry (Å, º)

**Table:**

*D*—H···*A*	*D*—H	H···*A*	*D*···*A*	*D*—H···*A*
N1—H1*A*···O1	0.93 (3)	1.96 (3)	2.869 (2)	167 (2)
N1—H1*B*···O1^i^	0.93 (3)	1.86 (3)	2.785 (2)	173 (2)
N1—H1*C*···O2^ii^	0.88 (3)	1.99 (3)	2.858 (2)	170 (2)

Symmetry codes: (i) −*x*+3/2, *y*−1/2, −*z*+1; (ii) *x*, *y*−1, *z*.

### 2-(Cyclohex-1-en-1-yl)ethan-1-aminium 4-bromobenzoate (VI) Crystal data

**Table:**

C_8_H_15_N^+^·C_7_H_4_BrO_2_^−^	*F*(000) = 668
*M**_r_* = 325.22	*D*_x_ = 1.426 Mg m^−^^3^
Monoclinic, *P*2_1_/*n*	Mo *K*α radiation, λ = 0.71073 Å
Hall symbol: -P 2yn	Cell parameters from 9914 reflections
*a* = 6.4391 (3) Å	θ = 3.2–28.3°
*b* = 17.0023 (8) Å	µ = 2.71 mm^−^^1^
*c* = 14.1588 (6) Å	*T* = 173 K
β = 102.241 (2)°	Rods, colourless
*V* = 1514.86 (12) Å^3^	0.68 × 0.18 × 0.1 mm
*Z* = 4	

### 2-(Cyclohex-1-en-1-yl)ethan-1-aminium 4-bromobenzoate (VI) Data collection

**Table:**

Bruker D8 Venture Photon CCD area detector diffractometer	3302 reflections with *I* > 2σ(*I*)
Graphite monochromator	*R*_int_ = 0.071
ω scans	θ_max_ = 28.0°, θ_min_ = 2.8°
Absorption correction: integration (XPREP; Bruker, 2016)	*h* = −8→8
*T*_min_ = 0.275, *T*_max_ = 0.776	*k* = −22→22
30862 measured reflections	*l* = −18→18
3658 independent reflections	

### 2-(Cyclohex-1-en-1-yl)ethan-1-aminium 4-bromobenzoate (VI) Refinement

**Table:**

Refinement on *F*^2^	0 constraints
Least-squares matrix: full	Hydrogen site location: mixed
*R*[*F*^2^ > 2σ(*F*^2^)] = 0.037	H atoms treated by a mixture of independent and constrained refinement
*wR*(*F*^2^) = 0.098	*w* = 1/[σ^2^(*F*_o_^2^) + (0.0477*P*)^2^ + 1.5945*P*] where *P* = (*F*_o_^2^ + 2*F*_c_^2^)/3
*S* = 1.05	(Δ/σ)_max_ = 0.001
3658 reflections	Δρ_max_ = 1.01 e Å^−^^3^
194 parameters	Δρ_min_ = −1.02 e Å^−^^3^
0 restraints	

### 2-(Cyclohex-1-en-1-yl)ethan-1-aminium 4-bromobenzoate (VI) Special details

**Table:**

Experimental. Numerical integration absorption corrections based on indexed crystal faces were applied using the XPREP routine (Bruker, 2016)
Geometry. All esds (except the esd in the dihedral angle between two l.s. planes) are estimated using the full covariance matrix. The cell esds are taken into account individually in the estimation of esds in distances, angles and torsion angles; correlations between esds in cell parameters are only used when they are defined by crystal symmetry. An approximate (isotropic) treatment of cell esds is used for estimating esds involving l.s. planes.

### 2-(Cyclohex-1-en-1-yl)ethan-1-aminium 4-bromobenzoate (VI) Fractional atomic coordinates and isotropic or equivalent isotropic displacement parameters (Å^2^)

**Table:**

	*x*	*y*	*z*	*U*_iso_*/*U*_eq_	Occ. (<1)
C1	0.9952 (4)	0.55176 (13)	0.35862 (16)	0.0297 (5)	
C2	1.2265 (4)	0.56748 (17)	0.38957 (19)	0.0394 (6)	
H2A	1.272267	0.599559	0.339287	0.047*	
H2B	1.303568	0.516784	0.393878	0.047*	
C3	1.2903 (4)	0.6097 (2)	0.4859 (2)	0.0482 (7)	
H3A	1.29862	0.571138	0.538886	0.058*	0.77 (2)
H3B	1.433308	0.632993	0.491224	0.058*	0.77 (2)
H3C	1.367424	0.571718	0.533684	0.058*	0.23 (2)
H3D	1.39227	0.651718	0.47853	0.058*	0.23 (2)
C4A	1.1367 (7)	0.6733 (4)	0.4972 (5)	0.0337 (12)	0.77 (2)
H4A	1.137787	0.714401	0.447738	0.04*	0.77 (2)
H4B	1.182544	0.697969	0.561611	0.04*	0.77 (2)
C4B	1.126 (3)	0.6445 (19)	0.5257 (16)	0.046 (5)	0.23 (2)
H4C	1.143892	0.622775	0.591802	0.055*	0.23 (2)
H4D	1.161962	0.701067	0.533582	0.055*	0.23 (2)
C5	0.9110 (4)	0.64165 (17)	0.4868 (2)	0.0432 (6)	
H5A	0.818606	0.655783	0.528143	0.052*	0.77 (2)
H5B	0.809547	0.672265	0.510406	0.052*	0.23 (2)
C6	0.8522 (4)	0.58524 (15)	0.40311 (17)	0.0337 (5)	
H6	0.706363	0.572658	0.380575	0.04*	
C7	0.9225 (4)	0.49832 (14)	0.27304 (16)	0.0342 (5)	
H7A	0.77838	0.478269	0.273552	0.041*	
H7B	1.019715	0.452701	0.277803	0.041*	
C8	0.9188 (4)	0.54181 (13)	0.17902 (15)	0.0278 (4)	
H8A	0.833004	0.590296	0.177594	0.033*	
H8B	1.065383	0.557518	0.176224	0.033*	
C9	0.2632 (3)	0.32298 (12)	0.19291 (14)	0.0213 (4)	
C10	0.4191 (3)	0.29872 (13)	0.27194 (15)	0.0253 (4)	
H10	0.559631	0.318645	0.279912	0.03*	
C11	0.3711 (3)	0.24585 (13)	0.33893 (15)	0.0271 (4)	
H11	0.477044	0.229697	0.392839	0.033*	
C12	0.1660 (3)	0.21718 (12)	0.32558 (15)	0.0240 (4)	
C13	0.0081 (3)	0.23954 (13)	0.24739 (16)	0.0278 (4)	
H13	−0.131731	0.218908	0.239255	0.033*	
C14	0.0588 (3)	0.29276 (13)	0.18123 (15)	0.0257 (4)	
H14	−0.047572	0.308672	0.127369	0.031*	
C15	0.3204 (3)	0.38114 (12)	0.12140 (15)	0.0237 (4)	
N1	0.8286 (3)	0.49309 (12)	0.09315 (13)	0.0242 (4)	
O1	0.5104 (3)	0.39903 (11)	0.13033 (13)	0.0366 (4)	
O2	0.1681 (3)	0.40722 (10)	0.05740 (12)	0.0327 (4)	
Br1	0.09846 (4)	0.14745 (2)	0.41940 (2)	0.03754 (10)	
H1A	0.716 (5)	0.4690 (18)	0.100 (2)	0.039 (8)*	
H1B	0.928 (5)	0.456 (2)	0.082 (2)	0.042 (8)*	
H1C	0.809 (5)	0.5244 (19)	0.045 (2)	0.042 (8)*	

### 2-(Cyclohex-1-en-1-yl)ethan-1-aminium 4-bromobenzoate (VI) Atomic displacement parameters (Å^2^)

**Table:**

	*U*^11^	*U*^22^	*U*^33^	*U*^12^	*U*^13^	*U*^23^
C1	0.0350 (11)	0.0303 (11)	0.0227 (10)	−0.0081 (9)	0.0036 (8)	0.0016 (8)
C2	0.0284 (11)	0.0489 (15)	0.0409 (13)	−0.0036 (10)	0.0075 (10)	−0.0104 (11)
C3	0.0314 (12)	0.0606 (18)	0.0491 (16)	−0.0057 (12)	0.0004 (11)	−0.0145 (14)
C4A	0.0330 (17)	0.037 (2)	0.030 (2)	−0.0094 (16)	0.0043 (15)	−0.0051 (17)
C4B	0.052 (8)	0.056 (13)	0.028 (8)	−0.010 (8)	0.006 (6)	−0.010 (7)
C5	0.0326 (12)	0.0538 (16)	0.0428 (14)	−0.0036 (11)	0.0072 (11)	−0.0164 (12)
C6	0.0263 (10)	0.0398 (12)	0.0339 (12)	−0.0060 (9)	0.0042 (9)	−0.0052 (10)
C7	0.0464 (13)	0.0322 (11)	0.0229 (10)	−0.0104 (10)	0.0049 (9)	0.0003 (9)
C8	0.0304 (11)	0.0277 (10)	0.0242 (10)	−0.0053 (8)	0.0037 (8)	0.0021 (8)
C9	0.0226 (9)	0.0218 (9)	0.0205 (9)	0.0003 (7)	0.0069 (7)	−0.0016 (7)
C10	0.0199 (9)	0.0289 (10)	0.0262 (10)	−0.0027 (8)	0.0029 (7)	−0.0005 (8)
C11	0.0250 (10)	0.0307 (11)	0.0232 (9)	0.0004 (8)	−0.0002 (8)	0.0029 (8)
C12	0.0267 (10)	0.0213 (9)	0.0249 (10)	−0.0002 (7)	0.0074 (8)	0.0032 (7)
C13	0.0203 (9)	0.0309 (11)	0.0316 (11)	−0.0030 (8)	0.0041 (8)	0.0059 (9)
C14	0.0218 (9)	0.0288 (10)	0.0248 (10)	−0.0007 (8)	0.0007 (7)	0.0041 (8)
C15	0.0279 (10)	0.0233 (9)	0.0223 (9)	−0.0006 (8)	0.0105 (8)	−0.0014 (7)
N1	0.0229 (8)	0.0297 (9)	0.0204 (8)	−0.0029 (7)	0.0055 (7)	0.0039 (7)
O1	0.0305 (8)	0.0442 (10)	0.0365 (9)	−0.0120 (7)	0.0101 (7)	0.0054 (7)
O2	0.0322 (8)	0.0390 (9)	0.0291 (8)	0.0059 (7)	0.0112 (6)	0.0137 (7)
Br1	0.03653 (15)	0.03802 (16)	0.03819 (15)	−0.00282 (9)	0.00818 (10)	0.01726 (10)

### 2-(Cyclohex-1-en-1-yl)ethan-1-aminium 4-bromobenzoate (VI) Geometric parameters (Å, º)

**Table:**

C1—C6	1.347 (3)	C7—H7A	0.99
C1—C2	1.485 (3)	C7—H7B	0.99
C1—C7	1.508 (3)	C8—N1	1.484 (3)
C2—C3	1.519 (4)	C8—H8A	0.99
C2—H2A	0.99	C8—H8B	0.99
C2—H2B	0.99	C9—C14	1.390 (3)
C3—C4B	1.429 (18)	C9—C10	1.398 (3)
C3—C4A	1.497 (6)	C9—C15	1.515 (3)
C3—H3A	0.99	C10—C11	1.388 (3)
C3—H3B	0.99	C10—H10	0.95
C3—H3C	0.99	C11—C12	1.383 (3)
C3—H3D	0.99	C11—H11	0.95
C4A—C5	1.527 (5)	C12—C13	1.388 (3)
C4A—H4A	0.99	C12—Br1	1.898 (2)
C4A—H4B	0.99	C13—C14	1.390 (3)
C4B—C5	1.377 (18)	C13—H13	0.95
C4B—H4C	0.99	C14—H14	0.95
C4B—H4D	0.99	C15—O1	1.241 (3)
C5—C6	1.509 (3)	C15—O2	1.266 (3)
C5—H5A	0.95	N1—H1A	0.86 (3)
C5—H5B	0.95	N1—H1B	0.94 (3)
C6—H6	0.95	N1—H1C	0.86 (3)
C7—C8	1.519 (3)		
			
C6—C1—C2	121.9 (2)	C5—C6—H6	118.2
C6—C1—C7	120.3 (2)	C1—C7—C8	110.83 (19)
C2—C1—C7	117.8 (2)	C1—C7—H7A	109.5
C1—C2—C3	114.4 (2)	C8—C7—H7A	109.5
C1—C2—H2A	108.7	C1—C7—H7B	109.5
C3—C2—H2A	108.7	C8—C7—H7B	109.5
C1—C2—H2B	108.7	H7A—C7—H7B	108.1
C3—C2—H2B	108.7	N1—C8—C7	112.16 (18)
H2A—C2—H2B	107.6	N1—C8—H8A	109.2
C4B—C3—C2	117.9 (7)	C7—C8—H8A	109.2
C4A—C3—C2	112.3 (3)	N1—C8—H8B	109.2
C4A—C3—H3A	109.2	C7—C8—H8B	109.2
C2—C3—H3A	109.2	H8A—C8—H8B	107.9
C4A—C3—H3B	109.2	C14—C9—C10	119.17 (19)
C2—C3—H3B	109.2	C14—C9—C15	121.33 (18)
H3A—C3—H3B	107.9	C10—C9—C15	119.49 (18)
C4B—C3—H3C	107.8	C11—C10—C9	120.78 (19)
C2—C3—H3C	107.8	C11—C10—H10	119.6
C4B—C3—H3D	107.8	C9—C10—H10	119.6
C2—C3—H3D	107.8	C12—C11—C10	118.66 (19)
H3C—C3—H3D	107.2	C12—C11—H11	120.7
C3—C4A—C5	111.8 (4)	C10—C11—H11	120.7
C3—C4A—H4A	109.3	C11—C12—C13	121.97 (19)
C5—C4A—H4A	109.3	C11—C12—Br1	118.58 (15)
C3—C4A—H4B	109.3	C13—C12—Br1	119.43 (16)
C5—C4A—H4B	109.3	C12—C13—C14	118.59 (19)
H4A—C4A—H4B	107.9	C12—C13—H13	120.7
C5—C4B—C3	126.4 (11)	C14—C13—H13	120.7
C5—C4B—H4C	105.7	C9—C14—C13	120.83 (19)
C3—C4B—H4C	105.7	C9—C14—H14	119.6
C5—C4B—H4D	105.7	C13—C14—H14	119.6
C3—C4B—H4D	105.7	O1—C15—O2	125.8 (2)
H4C—C4B—H4D	106.2	O1—C15—C9	117.77 (19)
C4B—C5—C6	113.6 (7)	O2—C15—C9	116.46 (18)
C6—C5—C4A	112.0 (3)	C8—N1—H1A	111 (2)
C6—C5—H5A	124	C8—N1—H1B	110.6 (18)
C4A—C5—H5A	124	H1A—N1—H1B	109 (3)
C4B—C5—H5B	123.2	C8—N1—H1C	106 (2)
C6—C5—H5B	123.2	H1A—N1—H1C	114 (3)
C1—C6—C5	123.6 (2)	H1B—N1—H1C	106 (3)
C1—C6—H6	118.2		
			
C6—C1—C2—C3	11.9 (4)	C14—C9—C10—C11	−0.8 (3)
C7—C1—C2—C3	−169.8 (2)	C15—C9—C10—C11	179.63 (19)
C1—C2—C3—C4B	−13.2 (17)	C9—C10—C11—C12	0.4 (3)
C1—C2—C3—C4A	−40.6 (5)	C10—C11—C12—C13	0.2 (3)
C2—C3—C4A—C5	57.2 (6)	C10—C11—C12—Br1	−178.05 (16)
C2—C3—C4B—C5	2 (4)	C11—C12—C13—C14	−0.4 (3)
C3—C4B—C5—C6	10 (4)	Br1—C12—C13—C14	177.80 (16)
C3—C4A—C5—C6	−43.8 (6)	C10—C9—C14—C13	0.5 (3)
C2—C1—C6—C5	0.6 (4)	C15—C9—C14—C13	−179.9 (2)
C7—C1—C6—C5	−177.7 (2)	C12—C13—C14—C9	0.1 (3)
C4B—C5—C6—C1	−11.7 (17)	C14—C9—C15—O1	−172.3 (2)
C4A—C5—C6—C1	15.5 (5)	C10—C9—C15—O1	7.3 (3)
C6—C1—C7—C8	100.0 (3)	C14—C9—C15—O2	7.7 (3)
C2—C1—C7—C8	−78.4 (3)	C10—C9—C15—O2	−172.70 (19)
C1—C7—C8—N1	−174.6 (2)		

### 2-(Cyclohex-1-en-1-yl)ethan-1-aminium 4-bromobenzoate (VI) Hydrogen-bond geometry (Å, º)

**Table:**

*D*—H···*A*	*D*—H	H···*A*	*D*···*A*	*D*—H···*A*
N1—H1*A*···O1	0.86 (3)	1.89 (3)	2.737 (2)	167 (3)
N1—H1*B*···O2^i^	0.94 (3)	1.85 (3)	2.763 (3)	164 (3)
N1—H1*C*···O2^ii^	0.86 (3)	1.89 (3)	2.727 (2)	167 (3)

Symmetry codes: (i) *x*+1, *y*, *z*; (ii) −*x*+1, −*y*+1, −*z*.

### (S)-1-Cyclohexylethan-1-aminium 4-bromobenzoate' (VII) Crystal data

**Table:**

C_8_H_18_N^+^·C_7_H_4_BrO_2_^−^	*F*(000) = 680
*M**_r_* = 328.24	*D*_x_ = 1.396 Mg m^−^^3^
Orthorhombic, *P*2_1_2_1_2_1_	Mo *K*α radiation, λ = 0.71073 Å
Hall symbol: P 2ac 2ab	Cell parameters from 9958 reflections
*a* = 6.2790 (3) Å	θ = 3.5–28.1°
*b* = 15.6610 (9) Å	µ = 2.63 mm^−^^1^
*c* = 15.8800 (8) Å	*T* = 173 K
*V* = 1561.57 (14) Å^3^	Needle, colourless
*Z* = 4	0.69 × 0.13 × 0.10 mm

### (S)-1-Cyclohexylethan-1-aminium 4-bromobenzoate' (VII) Data collection

**Table:**

Bruker D8 Venture Photon CCD area detector diffractometer	2687 reflections with *I* > 2σ(*I*)
Graphite monochromator	*R*_int_ = 0.068
ω scans	θ_max_ = 25.5°, θ_min_ = 2.9°
Absorption correction: integration (XPREP; Bruker, 2016)	*h* = −7→7
*T*_min_ = 0.452, *T*_max_ = 0.846	*k* = −18→18
21006 measured reflections	*l* = −19→19
2913 independent reflections	

### (S)-1-Cyclohexylethan-1-aminium 4-bromobenzoate' (VII) Refinement

**Table:**

Refinement on *F*^2^	Hydrogen site location: inferred from neighbouring sites
Least-squares matrix: full	H-atom parameters constrained
*R*[*F*^2^ > 2σ(*F*^2^)] = 0.078	*w* = 1/[σ^2^(*F*_o_^2^) + (0.1114*P*)^2^ + 6.6115*P*] where *P* = (*F*_o_^2^ + 2*F*_c_^2^)/3
*wR*(*F*^2^) = 0.211	(Δ/σ)_max_ < 0.001
*S* = 1.08	Δρ_max_ = 1.46 e Å^−^^3^
2913 reflections	Δρ_min_ = −0.48 e Å^−^^3^
174 parameters	Absolute structure: Flack *x* determined using 1026 quotients [(*I*^+^)-(*I*^-^)]/[(*I*^+^)+(*I*^-^)] (Parsons *et al.*, 2013)
0 restraints	Absolute structure parameter: 0.068 (9)
0 constraints	

### (S)-1-Cyclohexylethan-1-aminium 4-bromobenzoate' (VII) Special details

**Table:**

Experimental. Numerical integration absorption corrections based on indexed crystal faces were applied using the XPREP routine (Bruker, 2016)
Geometry. All esds (except the esd in the dihedral angle between two l.s. planes) are estimated using the full covariance matrix. The cell esds are taken into account individually in the estimation of esds in distances, angles and torsion angles; correlations between esds in cell parameters are only used when they are defined by crystal symmetry. An approximate (isotropic) treatment of cell esds is used for estimating esds involving l.s. planes.

### (S)-1-Cyclohexylethan-1-aminium 4-bromobenzoate' (VII) Fractional atomic coordinates and isotropic or equivalent isotropic displacement parameters (Å^2^)

**Table:**

	*x*	*y*	*z*	*U*_iso_*/*U*_eq_	
C1	0.773 (2)	0.0775 (8)	0.3199 (7)	0.053 (3)	
H1	0.740942	0.015006	0.317739	0.064*	
C2	0.612 (2)	0.1220 (11)	0.2688 (8)	0.068 (4)	
H2A	0.638924	0.184264	0.269256	0.082*	
H2B	0.467953	0.111721	0.292398	0.082*	
C3	0.624 (3)	0.0862 (13)	0.1743 (9)	0.084 (5)	
H3A	0.595663	0.024063	0.173633	0.1*	
H3B	0.516609	0.114896	0.138545	0.1*	
C4	0.844 (2)	0.1039 (11)	0.1413 (8)	0.077 (5)	
H4A	0.85316	0.08645	0.081516	0.093*	
H4B	0.873874	0.165911	0.144629	0.093*	
C5	1.005 (3)	0.0565 (12)	0.1911 (10)	0.083 (5)	
H5A	1.149674	0.066472	0.168707	0.1*	
H5B	0.974778	−0.005537	0.19016	0.1*	
C6	0.984 (3)	0.0910 (11)	0.2780 (10)	0.080 (5)	
H6A	1.013382	0.153146	0.276219	0.097*	
H6B	1.096015	0.064487	0.313464	0.097*	
C7	0.762 (2)	0.1051 (7)	0.4107 (7)	0.056 (3)	
H7	0.882202	0.075639	0.44015	0.067*	
C8	0.563 (3)	0.0786 (10)	0.4553 (8)	0.086 (6)	
H8A	0.468938	0.128083	0.461722	0.129*	
H8B	0.599286	0.05585	0.510964	0.129*	
H8C	0.490145	0.034387	0.422389	0.129*	
N1	0.7938 (13)	0.1998 (5)	0.4253 (4)	0.0331 (16)	
H1A	0.691137	0.229515	0.397416	0.05*	
H1B	0.924273	0.215652	0.40592	0.05*	
H1C	0.784863	0.21113	0.481408	0.05*	
C9	0.4242 (17)	0.3282 (6)	0.2666 (6)	0.036 (2)	
C10	0.631 (2)	0.3587 (7)	0.2719 (7)	0.051 (3)	
H10	0.690085	0.370093	0.325852	0.061*	
C11	0.7566 (19)	0.3734 (7)	0.2006 (6)	0.047 (2)	
H11	0.899884	0.392507	0.204207	0.057*	
C12	0.6544 (19)	0.3577 (6)	0.1225 (7)	0.046 (2)	
C13	0.4508 (18)	0.3286 (7)	0.1153 (7)	0.043 (2)	
H13	0.394001	0.31832	0.0608	0.052*	
C14	0.321 (2)	0.3131 (6)	0.1868 (6)	0.043 (2)	
H14	0.177757	0.294286	0.182502	0.052*	
C15	0.2958 (14)	0.3029 (6)	0.3460 (5)	0.0276 (18)	
O1	0.4108 (15)	0.2878 (6)	0.4104 (5)	0.061 (2)	
O2	0.1043 (14)	0.3044 (6)	0.3421 (5)	0.058 (2)	
Br1	0.8185 (2)	0.37723 (9)	0.02444 (6)	0.0586 (4)	

### (S)-1-Cyclohexylethan-1-aminium 4-bromobenzoate' (VII) Atomic displacement parameters (Å^2^)

**Table:**

	*U*^11^	*U*^22^	*U*^33^	*U*^12^	*U*^13^	*U*^23^
C1	0.059 (7)	0.061 (6)	0.040 (6)	−0.010 (6)	0.009 (5)	−0.008 (5)
C2	0.067 (8)	0.080 (8)	0.058 (7)	0.024 (8)	0.014 (6)	−0.014 (7)
C3	0.083 (10)	0.126 (14)	0.043 (7)	0.023 (9)	−0.018 (7)	−0.023 (8)
C4	0.080 (9)	0.110 (12)	0.042 (6)	0.018 (9)	0.026 (7)	0.019 (7)
C5	0.083 (11)	0.104 (12)	0.063 (9)	0.005 (10)	−0.005 (8)	−0.020 (9)
C6	0.068 (9)	0.098 (11)	0.076 (10)	−0.020 (8)	0.018 (8)	−0.044 (8)
C7	0.081 (9)	0.047 (6)	0.039 (6)	−0.012 (5)	−0.009 (5)	−0.004 (5)
C8	0.137 (15)	0.080 (9)	0.042 (7)	−0.059 (10)	0.017 (8)	−0.014 (6)
N1	0.032 (4)	0.050 (4)	0.018 (3)	0.002 (4)	−0.004 (3)	0.001 (3)
C9	0.040 (5)	0.040 (5)	0.028 (5)	−0.008 (4)	0.003 (4)	0.004 (4)
C10	0.074 (8)	0.046 (6)	0.033 (5)	0.002 (5)	0.004 (5)	0.002 (4)
C11	0.070 (7)	0.045 (5)	0.027 (4)	−0.010 (5)	0.003 (4)	0.001 (4)
C12	0.055 (6)	0.044 (5)	0.038 (5)	0.007 (5)	0.011 (5)	−0.001 (4)
C13	0.049 (6)	0.048 (6)	0.032 (5)	0.003 (5)	−0.003 (5)	−0.002 (4)
C14	0.066 (7)	0.036 (4)	0.026 (5)	0.001 (5)	−0.005 (5)	0.006 (4)
C15	0.025 (4)	0.040 (4)	0.018 (4)	0.005 (4)	0.006 (3)	0.001 (3)
O1	0.059 (5)	0.093 (6)	0.031 (4)	0.015 (5)	0.007 (4)	0.008 (4)
O2	0.048 (5)	0.082 (6)	0.044 (5)	−0.001 (4)	0.010 (4)	0.013 (4)
Br1	0.0598 (7)	0.0877 (8)	0.0283 (5)	0.0091 (7)	0.0127 (5)	0.0065 (5)

### (S)-1-Cyclohexylethan-1-aminium 4-bromobenzoate' (VII) Geometric parameters (Å, º)

**Table:**

C1—C2	1.475 (19)	C8—H8A	0.98
C1—C6	1.498 (19)	C8—H8B	0.98
C1—C7	1.507 (16)	C8—H8C	0.98
C1—H1	1	N1—H1A	0.91
C2—C3	1.604 (18)	N1—H1B	0.91
C2—H2A	0.99	N1—H1C	0.91
C2—H2B	0.99	C9—C10	1.389 (17)
C3—C4	1.50 (2)	C9—C14	1.442 (14)
C3—H3A	0.99	C9—C15	1.548 (12)
C3—H3B	0.99	C10—C11	1.397 (15)
C4—C5	1.48 (2)	C10—H10	0.95
C4—H4A	0.99	C11—C12	1.418 (15)
C4—H4B	0.99	C11—H11	0.95
C5—C6	1.49 (2)	C12—C13	1.362 (17)
C5—H5A	0.99	C12—Br1	1.892 (11)
C5—H5B	0.99	C13—C14	1.418 (15)
C6—H6A	0.99	C13—H13	0.95
C6—H6B	0.99	C14—H14	0.95
C7—C8	1.50 (2)	C15—O2	1.204 (12)
C7—N1	1.515 (13)	C15—O1	1.273 (12)
C7—H7	1		
			
C2—C1—C6	107.4 (12)	C1—C7—N1	114.9 (9)
C2—C1—C7	111.0 (11)	C8—C7—H7	106.3
C6—C1—C7	115.2 (11)	C1—C7—H7	106.3
C2—C1—H1	107.7	N1—C7—H7	106.3
C6—C1—H1	107.7	C7—C8—H8A	109.5
C7—C1—H1	107.7	C7—C8—H8B	109.5
C1—C2—C3	108.4 (11)	H8A—C8—H8B	109.5
C1—C2—H2A	110	C7—C8—H8C	109.5
C3—C2—H2A	110	H8A—C8—H8C	109.5
C1—C2—H2B	110	H8B—C8—H8C	109.5
C3—C2—H2B	110	C7—N1—H1A	109.5
H2A—C2—H2B	108.4	C7—N1—H1B	109.5
C4—C3—C2	107.9 (13)	H1A—N1—H1B	109.5
C4—C3—H3A	110.1	C7—N1—H1C	109.5
C2—C3—H3A	110.1	H1A—N1—H1C	109.5
C4—C3—H3B	110.1	H1B—N1—H1C	109.5
C2—C3—H3B	110.1	C10—C9—C14	122.0 (10)
H3A—C3—H3B	108.4	C10—C9—C15	121.8 (9)
C5—C4—C3	110.3 (13)	C14—C9—C15	116.1 (9)
C5—C4—H4A	109.6	C9—C10—C11	122.3 (11)
C3—C4—H4A	109.6	C9—C10—H10	118.8
C5—C4—H4B	109.6	C11—C10—H10	118.8
C3—C4—H4B	109.6	C10—C11—C12	115.2 (11)
H4A—C4—H4B	108.1	C10—C11—H11	122.4
C4—C5—C6	104.8 (15)	C12—C11—H11	122.4
C4—C5—H5A	110.8	C13—C12—C11	123.8 (10)
C6—C5—H5A	110.8	C13—C12—Br1	119.8 (9)
C4—C5—H5B	110.8	C11—C12—Br1	116.5 (9)
C6—C5—H5B	110.8	C12—C13—C14	121.9 (10)
H5A—C5—H5B	108.9	C12—C13—H13	119
C5—C6—C1	115.8 (13)	C14—C13—H13	119
C5—C6—H6A	108.3	C13—C14—C9	114.7 (11)
C1—C6—H6A	108.3	C13—C14—H14	122.6
C5—C6—H6B	108.3	C9—C14—H14	122.6
C1—C6—H6B	108.3	O2—C15—O1	127.7 (9)
H6A—C6—H6B	107.4	O2—C15—C9	118.2 (8)
C8—C7—C1	114.4 (11)	O1—C15—C9	114.0 (8)
C8—C7—N1	108.0 (11)		
			
C6—C1—C2—C3	56.2 (16)	C15—C9—C10—C11	173.2 (10)
C7—C1—C2—C3	−177.1 (13)	C9—C10—C11—C12	2.1 (16)
C1—C2—C3—C4	−60.0 (18)	C10—C11—C12—C13	−1.5 (16)
C2—C3—C4—C5	63.5 (19)	C10—C11—C12—Br1	179.8 (8)
C3—C4—C5—C6	−62.2 (18)	C11—C12—C13—C14	1.4 (17)
C4—C5—C6—C1	62 (2)	Br1—C12—C13—C14	−179.9 (8)
C2—C1—C6—C5	−61.5 (19)	C12—C13—C14—C9	−1.8 (15)
C7—C1—C6—C5	174.3 (14)	C10—C9—C14—C13	2.5 (14)
C2—C1—C7—C8	67.0 (16)	C15—C9—C14—C13	−173.7 (9)
C6—C1—C7—C8	−170.7 (15)	C10—C9—C15—O2	155.7 (11)
C2—C1—C7—N1	−58.7 (15)	C14—C9—C15—O2	−28.1 (14)
C6—C1—C7—N1	63.5 (17)	C10—C9—C15—O1	−20.1 (14)
C14—C9—C10—C11	−2.8 (17)	C14—C9—C15—O1	156.1 (9)

### (S)-1-Cyclohexylethan-1-aminium 4-bromobenzoate' (VII) Hydrogen-bond geometry (Å, º)

**Table:**

*D*—H···*A*	*D*—H	H···*A*	*D*···*A*	*D*—H···*A*
N1—H1*A*···O1	0.91	1.99	2.781 (12)	144
N1—H1*B*···O2^i^	0.91	2.06	2.870 (12)	148
N1—H1*C*···O1^ii^	0.91	1.89	2.718 (10)	150

Symmetry codes: (i) *x*+1, *y*, *z*; (ii) *x*+1/2, −*y*+1/2, −*z*+1.
